# Parkinson's disease: pathogenesis and therapeutic strategies

**DOI:** 10.1186/s43556-026-00445-0

**Published:** 2026-04-08

**Authors:** Shanshan Zhang, Tingyu Wang, Ye Peng, Qianchen Wang, Zhao Zhang, Shifeng Chu, Hefei Huang, Naihong Chen

**Affiliations:** 1 The First College of Clinical Medical Science, China Three Gorges University, Yichang, Hubei 443000 China; 2 China Three Gorges University College of Medicine and Health Sciences, Yichang, 443002 China; 3 Department of Pharmacy, Hunan University of Chinese Medicine, Changsha, 410208 China; 4 Department of Pharmacy, The First Affiliated Hospital of China Medical University, Shenyang, Liaoning 110001 China; 5 State Key Laboratory of Bioactive Substances and Functions of Natural Medicines, Institute of Materia Medica & Neuroscience Center, Chinese Academy of Medical Sciences and Peking Union Medical College, Beijing, 100050 China

**Keywords:** Parkinson’s disease, α-synuclein, Pathogenesis, Medication, Treatment progress

## Abstract

Parkinson's disease (PD) is a neurodegenerative disorder primarily characterized by motor impairments such as bradykinesia, tremor, and rigidity. Its neuropathological hallmarks include the progressive loss of dopaminergic neurons in the substantia nigra pars compacta (SNc) and the aggregation of α-synuclein (α-syn) into Lewy bodies (LBs), which gradually spread to other central nervous system regions and peripheral tissues. The etiology of PD is multifactorial, involving a complex interplay of genetic predisposition, aging, environmental exposures, and lifestyle factors. Disease pathogenesis is predominantly driven by the synergistic action of core pathological mechanisms, including α-syn aggregation, oxidative stress (OS), mitochondrial dysfunction, and neuroinflammation. In recent years, the role of peripheral-central communication pathways in disease initiation and propagation has garnered significant attention. To date, pharmacotherapy remains the mainstay for improving the quality of life in PD patients. The most commonly used clinical agents primarily target the replenishment of depleted dopamine in the brain. However, these medications only alleviate symptoms and do not slow disease progression. This inherent limitation underscores the urgent need for more effective therapeutic strategies. This review aims to systematically outline the network of PD pathogenesis and the evolution of its treatment strategies, with a particular emphasis on a holistic perspective from etiology to therapy. It critically evaluates current bottlenecks in drug treatment and provides an in-depth analysis of potential candidate drugs for PD, as well as the latest advances in α-syn-targeted immunotherapies, iPSC-based regenerative therapies, and gene therapies. Building on this foundation, we further argue that the future of PD management must shift towards integrated, multi-target, and personalized therapeutic strategies to overcome existing efficacy barriers.

## Introduction

Parkinson's disease is the second most common neurodegenerative disorder after Alzheimer's disease. The average age of onset is around 60 years. With the aging of the global population, the incidence of PD is gradually increasing worldwide. Currently, approximately 1%−2% of people aged over 60 are affected by this disease [1]. The number of PD patients is projected to rise from 7 million in 2015 to 13 million by 2040 [2]. The pathological hallmarks of PD include the misfolding and aggregation of α-syn into LBs, leading to the degeneration of dopaminergic neurons in the substantia nigra pars compacta and the depletion of striatal dopamine. Its clinical manifestations are primarily motor dysfunctions and various non-motor symptoms [3]. As the disease progresses, all clinical symptoms worsen to varying degrees, imposing a significant burden on families and society [4].

As the precise pathological mechanisms of PD remain incompletely understood, current treatments mainly rely on pharmacological modulation of neurotransmitters in the brain. Dopamine replacement therapies such as levodopa (L-DOPA) remain the mainstream clinical treatment for PD [5]. However, these therapeutic approaches are primarily limited to symptom control and cannot delay or halt disease progression. Therefore, exploring novel therapeutic strategies for PD in the context of unclear etiology and pathogenesis presents a significant challenge for future anti-PD drug development.

In recent years, important advancements have been made in PD research. On the mechanistic level [6], investigations have expanded beyond traditional core pathways such as OS, mitochondrial dysfunction, and neuroinflammation to encompass complex networks involving multi-organ system interactions like the gut-brain axis and spleen-brain axis. This has revealed new hypotheses suggesting that α-syn may propagate from the periphery to the central nervous system along neural pathways [7]. On the therapeutic front, alongside novel drug delivery strategies like levodopa inhalation powder and continuous subcutaneous infusions [8], disease-modifying therapies targeting specific molecules such as α-syn and c-Abl, as well as cutting-edge approaches like immunotherapy, stem cell therapy, and gene therapy, have shown considerable potential. Against this backdrop, this review aims to provide a comprehensive and in-depth synthesis of the pathogenesis and therapeutic strategies for PD, hoping to offer insights for future research into PD mechanisms and the development of treatments and interventions.

## The etiology of Parkinson’s disease

The etiology of PD remains unclear and may involve factors such as genetics, environment, gender, age, and lifestyle [9]. With advancing age, the incidence of PD gradually increases, and aging constitutes the most significant risk factor for the disease [10]. Compared with females, males exhibit a higher incidence of PD [11]. Additionally, studies have found that gut microbiota plays an important role in regulating motor dysfunction [12]. Metabolites produced by gut microbiota may influence brain neurons, and PD patients often experience gastrointestinal dysfunction. Therefore, alterations in the gut microbiota may represent a risk factor for the development of PD.

### Genetic factor

Genetic factors represent a common cause of PD, with a substantial proportion of PD cases influenced by genetic components [13]. In 1997, the first PD-associated gene, SNCA, was identified in an Italian family. It is an autosomal dominant gene located on human chromosome 4q21-23, containing six exons and encoding a 140-amino acid protein. SNCA is involved in encoding α-syn [14]. Since the discovery of SNCA, approximately 100 different genes or genetic loci have been found to be associated with PD to date [15]. Among these, high-penetrance monogenic forms of PD mainly include SNCA, VPS35, PRKN/PARK7/PINK1, while genes with variable penetrance primarily include LRRK2 and GBA [16]. Interactions between many common and rare genetic variants may also contribute to PD.

Patients with SNCA gene mutations are typically younger, experience rapid disease progression, exhibit swiftly advancing motor symptoms alongside prominent non-motor symptoms, and demonstrate a decline in cognitive ability within a short period [17]. VPS35 gene mutations can lead to mitochondrial dysfunction and autophagy defects, preventing the timely clearance of aggregated proteins and thereby promoting Lewy body formation, followed by damage to dopaminergic (DA) neurons [18]. PRKN, PINK1, and PARK7 are common autosomal recessive genes. Mutations in these genes result in impaired mitophagy and inflammatory responses, leading to the accumulation of abnormally aggregated proteins, exacerbating neuronal damage, and causing peripheral neuropathy and autonomic dysfunction. This manifests as gait and dystonia, tremor, and ataxia. However, PD caused by these genetic mutations usually progresses relatively slowly [19]. LRRK2 gene mutations are autosomal dominant. Mutated LRRK2 impairs vesicle trafficking, mitochondrial function, and autophagy, leading to significant α-syn accumulation and degeneration of nigral DA neurons. Patients present with symptoms such as tremor, dystonia, bradykinesia, cognitive decline, and psychiatric abnormalities [20]. GBA gene mutations cause dysfunction of the autophagy-lysosome pathway and reduced glucocerebrosidase (GCase) activity [21], resulting in α-syn aggregation and OS. This is followed by loss of nigrostriatal dopaminergic neurons, with patients exhibiting manifestations such as depression, sleep disturbances, and cognitive dysfunction [22].

Genetically caused PD is primarily divided into two forms: familial PD and sporadic PD. Familial PD, often referred to as Mendelian or monogenic PD, is generally rare but plays a crucial role in PD development due to its high penetrance. Genome-wide association studies (GWAS) have found that sporadic PD is believed to arise from interactions between the genome and the environment and is strongly associated with common genetic variants exhibiting low-penetrance effects [23]. Genetic variants account for approximately 25% of the risk factors for PD, and individuals with a family history of PD have a higher probability of developing the disease [24].

### Environment and lifestyle

Mounting evidence suggests that environmental factors may contribute significantly to the prevalence of PD [25]. For instance, pesticides, organic solvents, agricultural work, well-water consumption, air pollution, traumatic head injury, and type 2 diabetes have been closely associated with the development of PD [11]. Furthermore, alterations in metal ion homeostasis within the brain also play a crucial role in PD pathogenesis [26]. Pesticides such as paraquat and rotenone can impair vesicular transport, neuronal function, lysosomal activity, and mitochondrial integrity. Long-term exposure to these agents may elevate the risk of PD [27].

MPTP, a well-established neurotoxin used in modeling PD, induces OS, aberrant α-syn aggregation, and dopaminergic neuronal death by inhibiting mitochondrial complex I [28]. This highlights the potential of environmental toxins to trigger PD through similar mechanisms. Research has shown that PM2.5 generated from biomass fuel activates the IL-17A/IL-17RA signaling pathway [29], leading to systemic and cerebral neuroinflammation, ultimately resulting in dopaminergic neuronal loss, α-syn pathology, and Parkinsonian motor and cognitive deficits [30]. Additionally, metabolic dysregulation of metal ions such as iron, copper, and manganese in the brain can catalyze Fenton reactions, generating reactive oxygen species that exacerbate OS and protein misfolding [31]. Interestingly, coffee and tea consumption have been found to potentially decelerate PD progression. Caffeine in coffee may exert neuroprotective effects by blocking adenosine A2A receptors, thereby promoting dopaminergic neurotransmission. Tea, likely due to its antioxidant components, may also offer protective benefits to the organism [32].

### Gut-Brain axis and microbiota in PD pathogenesis

In recent years, accumulating evidence suggests that the gut microbiota may play a significant role in the progression of PD. As the gastrointestinal tract is in direct contact with the environment, various environmental toxins can also impact the gut microbiome. PD patients often exhibit gut dysbiosis, characterized by an increase in Akkermansia and decreases in Faecalibacterium and Roseburia [33]. Such microbial imbalance may lead to impaired intestinal barrier function, inflammatory responses, and abnormal aggregation of α-syn within the enteric nervous system. This pathology can subsequently propagate to the central nervous system via the vagus nerve, potentially triggering PD [34].

The bidirectional communication pathway between the gut and the brain has long been a focus of research. A potential connection exists between gut bacteria and the brain, a bidirectional interaction referred to as the microbiota-gut-brain axis [35]. This axis comprises the central nervous system, autonomic nervous system, enteric nervous system, and the hypothalamic–pituitary–adrenal axis. Signals from the gastrointestinal tract can reach the central nervous system, while the central nervous system can relay signals back to the enteric nervous system [36]. This system serves as a critical interface for the interaction between microbiota and brain function [37].

Research by Challis et al. demonstrated that inoculation of pathogenic α-syn preformed fibrils into the gut of aged mice resulted in a marked increase of α-syn in the brainstem and the emergence of pathological changes in the central nervous system [38]. This indicates that α-syn can propagate from the gut to the brain. Conversely, injection of α-syn into the striatum also induced pathological manifestations within the enteric nervous system, demonstrating that α-syn propagation can occur not only from the gut to the brain but also from the brain back to the gut. These findings underscore the important role of the microbiota-gut-brain axis in the pathogenesis of PD.

### Role of peripheral organs: spleen, liver, and skin

As a vital immune organ, the spleen has also been identified as a potential therapeutic target for neurodegenerative diseases. It influences central nervous system inflammation by regulating peripheral immune responses [39]. Research indicates that spleen dysregulation can activate immune cells such as M1 macrophages. Once activated, these macrophages promote the release of pro-inflammatory factors like tumor necrosis factor-α (TNF-α), activate signaling pathways including NF-κB, and drive neuroinflammation throughout the body and brain [40]. This process exacerbates dopaminergic neuron damage, accelerating the progression of PD [41].

Recent research has shown that the liver, a major detoxification organ, can also accumulate α-syn. Populations with hepatic α-syn accumulation exhibit a higher incidence of PD [42]. Thus, α-syn deposition in the liver may also contribute to PD pathogenesis. To date, multiple studies have observed deposits of α-syn and phosphorylated α-syn in skin biopsies from PD patients [43], with varying α-syn positivity rates across different biopsy sites [44]. Therefore, α-syn aggregation in the skin may play a significant role in PD development.

These peripheral organ–brain axis mechanisms collectively suggest that α-syn may originate in the periphery and propagate to the central nervous system through multiple pathways, thereby driving PD pathology. Intervention strategies targeting these axes—such as modulating gut microbiota, peripheral immunity, or hepatic metabolism—may offer novel directions for disease-modifying therapies in PD.

## Pathogenesis of Parkinson's disease

**Fig. 1:**
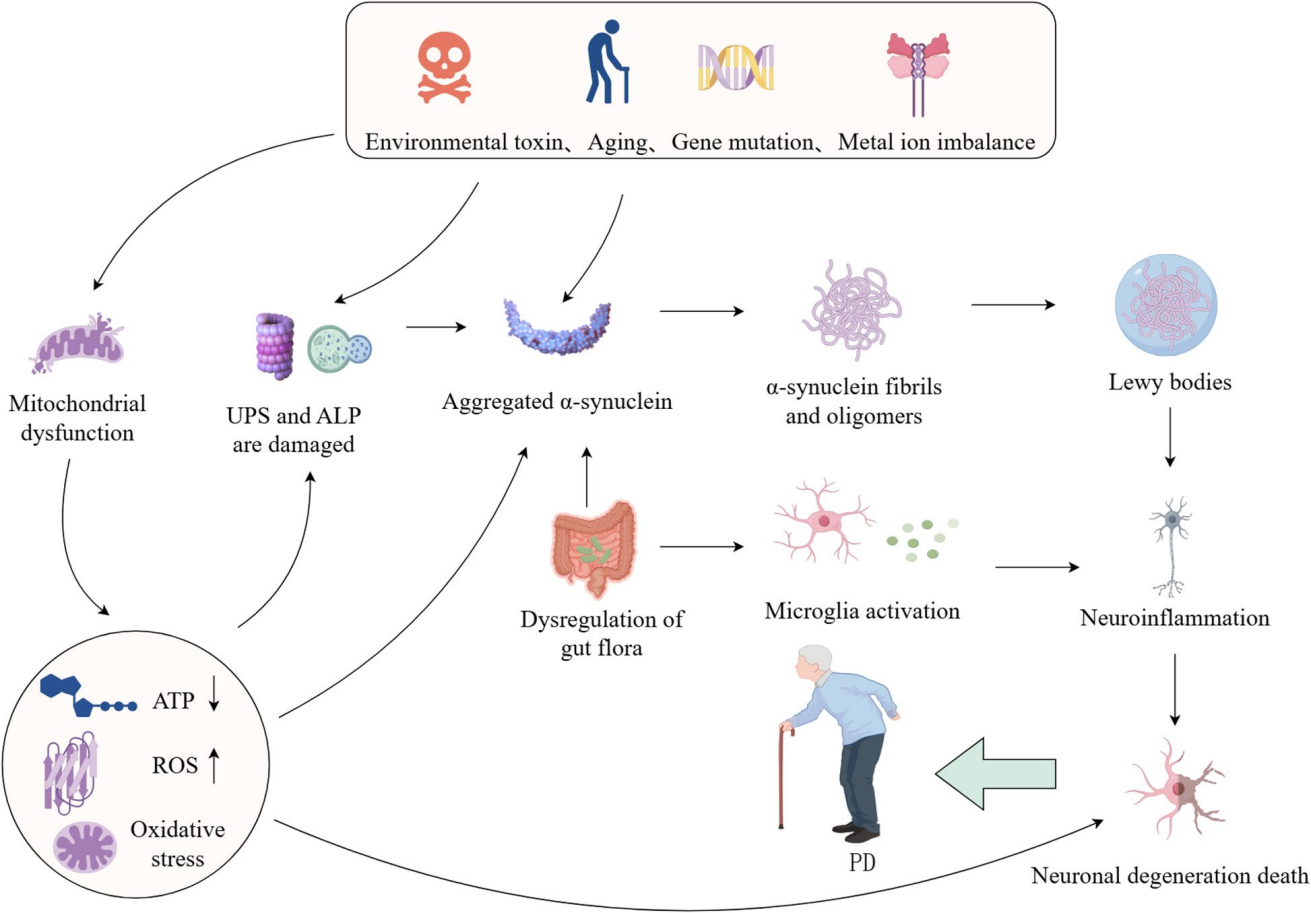
Molecular mechanisms contributing to Parkinson's disease. The figure illustrates the interplay between the diverse pathogenic mechanisms of PD and their contribution to the disease's progression. A number of factors, including environmental toxins, the process of aging, genetic mutations, imbalances in metal ions, and others, can contribute to the development of mitochondrial dysfunction, damage to the ubiquitin–proteasome system (UPS) and autophagy-lysosome system, and the aggregation of α-syn; Mitochondrial dysfunction results in a reduction in adenosine triphosphate (ATP) synthesis, an increase in ROS production and OS. This, in turn, leads to damage to the UPS and autophagy-lysosomal system, as well as the aggregation of α-syn, denaturation of neurons and their subsequent death. Intestinal dysbacteriosis has been demonstrated to cause α-syn aggregation and microglia activation, which in turn induces neuroinflammation. The formation of α-syn aggregates into fibrils and LBs has been linked to the onset of neuroinflammation, which in turn leads to neuronal deformation and death. This process plays a pivotal role in the development of PD

The pathogenesis of PD arises from a highly integrated network rather than being driven by a single linear pathway. This network centers on the abnormal aggregation of α-syn as its core driver, which is deeply interconnected with major pathological modules including mitochondrial dysfunction, OS, neuroinflammation, and metabolic disturbances [45]. Specifically, α-syn pathology not only directly impairs mitochondrial function and activates neuroinflammation, but its own aggregation process is also markedly promoted by OS and inflammatory environments [46]. In turn, dysfunctional mitochondria generate excessive reactive oxygen species (ROS), which can directly induce neuronal damage and further drive the cascade of α-syn misfolding and neuroinflammatory amplification. Concurrently, pathological signals originating from peripheral systems such as the gut–brain axis and spleen–brain axis continuously propagate to the central nervous system, persistently exacerbating the pathological processes within the central network and ultimately leading to the progressive loss of nigral dopaminergic neurons [3]. A deep understanding of the pathogenic mechanisms of PD is of crucial importance for overcoming the limitations of current single-target therapies and for developing synergistic treatment strategies capable of simultaneously intervening at multiple key nodes (Fig. 1).

### Abnormal aggregation of proteins

Α-syn is a soluble, small-molecule protein consisting of 140 amino acids encoded by the SNCA gene, primarily localized at the presynaptic terminals of neurons. α-syn primarily consists of three domains: the N-terminus, the central hydrophobic NAC region, and the C-terminus. The N-terminus mainly maintains the helical structure of α-syn, and familial PD-associated mutations in the SNCA gene frequently occur in this region. The NAC region is the central domain of α-syn, where the conformational transition from α-helix to β-sheet structures primarily occurs, playing a crucial role in PD pathogenesis [47]. Under physiological conditions, α-syn exhibits no neurotoxicity. However, pathological states such as OS, genetic mutations, and iron overload can trigger abnormal accumulation of α-syn. Mutations and overexpression of the α-syn gene induce conformational changes, transforming soluble monomers into insoluble toxic aggregates. These aggregates further aggregate into more toxic fibrillar forms, ultimately forming LBs.

Notably, α-syn pathology is not an isolated process; it is both a consequence of other mechanisms and an active driver of disease progression. α-syn aggregates can spread within the central nervous system and exert cytotoxicity through multiple pathways, such as mitochondrial dysfunction, endoplasmic reticulum stress, and synaptic damage. This alters neuronal membrane potential and OS states, ultimately leading to neuronal death [23, 48, 49]. Additionally, PD pathogenesis primarily involves vesicular transport and the maintenance of lysosomes and mitochondria [50]. α-syn oligomers can damage mitochondrial complex I, impairing respiratory function, inducing selective oxidation of ATP synthase and mitochondrial lipid peroxidation, leading to mitochondrial dysfunction and subsequent cell death [51].

With aging, the functions of the UPS and autophagy-lysosomal pathway gradually decline, leading to a significant increase in α-syn expression in the substantia nigra region and resulting in metabolic disorders [52]. In PD patients, massive accumulation of α-syn in the brain forms LBs. Misfolded α-syn exhibits properties similar to prions. As the disease progresses, LBs proliferate within the brain and migrate to other regions, causing multi-regional brain degeneration while simultaneously activating the NLRP3 inflammasome to trigger neuroinflammation [53].

### Oxidative stress

Oxidative stress plays a critical role in the progression of PD. In current research, one of the most important pathogenic mechanisms of PD is the DA neurons caused by OS. During aging, OS represents a primary process that directly impairs the central nervous system [54]. ROS are highly reactive molecules mainly involved in various redox reactions in the body and serve important defensive functions. However, excessive generation of ROS can lead to OS [55].

Under normal physiological conditions, excess free radicals produced by brain metabolism can be cleared by endogenous antioxidants such as superoxide dismutase (SOD) and glutathione (GSH), maintaining a balance between ROS production and elimination [56]. In PD patients, the levels of antioxidants in the brain are reduced, and both the activity of antioxidant enzymes and the capacity to scavenge free radicals are diminished. This disrupts the body's redox balance, triggering OS. Consequently, free radicals accumulate substantially in the substantia nigra, and cytotoxic substances that cannot be cleared aggregate within neurons, ultimately leading to cell death and DA neurons [57].

Additionally, when aged cells excessively accumulate in the body, the release of highly reactive molecules such as reactive oxygen species and reactive nitrogen species (RNS) increases, inducing OS. This impairs cellular functions and further exacerbates mitochondrial dysfunction [58]. Importantly, OS engages in profound bidirectional interactions with other pathogenic mechanisms. On one hand, ROS can directly modify α-syn, accelerating its abnormal aggregation; on the other hand, it is both a major product of mitochondrial dysfunction and a contributor to further mitochondrial damage [59].

### Mitochondrial dysfunction

Mitochondria serve as the primary intracellular source of ROS during normal aging. The ATP generated by mitochondria powers neural activity through oxidative phosphorylation in the mitochondrial electron transport chain and helps maintain intracellular homeostasis. Mitochondrial dysfunction arises from the combined effects of environmental and genetic factors and is closely linked to the onset of PD [60]. Such dysfunction leads to altered mitochondrial dynamics, impairment of the mitochondrial electron transport chain, increased ROS production, and consequently exacerbates OS [61]. This disrupts cellular energy metabolism, reduces ATP synthesis, and results in progressive cellular dysfunction and neurodegeneration. ROS derived from mitochondria can also impair lysosomal and proteasomal function and promote abnormal aggregation of α-syn [62].

Moreover, the balance of metal ions in the body plays a vital role in sustaining normal neurophysiological activity. With advancing age, certain changes in metal ion levels occur, and alterations in the content of metal ions in the brains of PD patients contribute to disease pathogenesis. Studies indicate that elevated levels of metals such as Fe, Cu, Mn, and Zn can damage the UPS and are associated with OS, mitochondrial dysfunction, and protein misfolding [63]. Furthermore, redox-active metals like copper and iron can catalyze the Fenton reaction, generating substantial amounts of ROS, which in turn induces OS and ultimately leads to cell death. Increased ROS also promotes iron oxidation, producing cytotoxic hydroxyl radicals from hydrogen peroxide and aggravating α-syn aggregation. Research shows that elevated manganese levels enhance intracellular ROS formation, inhibit mitochondrial complexes I and II of the electron transport chain, disrupt mitochondrial function, and cause dopaminergic cell death [64].

Additionally, Zn^2^⁺ accumulation promotes the death of dopaminergic neurons, and Zn^2^⁺ deposition has been observed in degenerating dopaminergic neurons in PD mouse models [65]. Thus, imbalance in metal ions may facilitate the progression of PD. Meanwhile, mitochondrial dysfunction impairs ATP-dependent lysosomal and proteasomal functions, weakens the cellular self-clearing capacity, and thereby indirectly contributes to α-syn accumulation. Consequently, mitochondria are not only a source of energy but also a central hub connecting various pathogenic pathways [66].

### Neuroinflammation

Neuroinflammation is a critical factor in the pathogenesis of PD. Although neuroinflammation serves as a neuroprotective mechanism, prolonged neuroinflammation can lead to neurotoxicity and subsequent neurodegeneration. Chronic inflammation directly or indirectly promotes the progression of PD. Under normal physiological conditions, microglia and astrocytes maintain homeostasis in the central nervous system by releasing neurotrophic factors and synaptic glutamate [67]. Under pathological stimulation, overactivation of microglia leads to the release of large amounts of pro-inflammatory factors, ROS, and nitric oxide (NO), triggering uncontrolled inflammatory responses that ultimately result in neuronal damage and sustained neuroinflammation [68].

Numerous studies have shown that, particularly in microglia, the activation of inflammasomes induces chronic neuroinflammation, which in turn initiates a cascade of reactions that damage dopaminergic neurons, leading to PD-like symptoms. Concurrently, activation of the NLRP3 inflammasome has been observed in Parkinson's patients, suggesting that inflammasomes play a significant role in neuroinflammation [69, 70]. In the brains of PD patients, levels of microglia and astrocytes are abnormally elevated. Activated glial cells release various chemokines and inflammatory mediators, including interleukin-1β (IL-1β), IL-4, IL-6, TNF-α, and interferon-β, which disrupt the blood–brain barrier. These pro-inflammatory factors stimulate astrocytes, contributing to the degeneration and death of dopaminergic neurons and oligodendrocytes [71]. Inflammatory cytokines not only directly damage neurons but also further impair mitochondrial function, inhibit proteasome activity, and create a favorable environment for α-syn aggregation, thereby exacerbating proteostasis imbalance and OS [72].

### Disturbance of metabolism

Recent studies have indicated that glucose metabolism, lipid metabolism, and amino acid metabolism are closely associated with the development of PD. With aging, reduced cellular metabolic activity leads to the accumulation of ROS, resulting in abnormal protein aggregation and organelle damage, which ultimately cause UPS dysfunction and impaired autophagy. These processes promote cell death, neuronal loss, and subsequently trigger PD [49, 73].

Abnormal glucose metabolism is closely associated with PD. Compared to healthy individuals, PD patients exhibit a higher prevalence of diabetes. Furthermore, an increased risk of PD onset has been observed in patients with hyperglycemia and diabetes [74]. Hyperglycemia can impair PD-related mesolimbic cortical pathways and nigrostriatal motor pathways, with the nigrostriatal motor pathway undergoing preferential degeneration [75]. Research indicates that hyperglycemia exacerbates PD pathology through multiple mechanisms. On the one hand, it induces OS by upregulating the transcription factor Nuclear Factor E2-related Factor 2 (Nrf2) and downregulating its inhibitor Keap1 [76], while also triggering neuroinflammation that causes OS and mitochondrial dysfunction in dopaminergic neurons [77]. On the other hand, it promotes α-syn aggregation by increasing thioredoxin-interacting protein (TXNIP), thereby inhibiting autophagy flux and mitochondrial autophagy, which accelerates dopaminergic neuron apoptosis and worsens PD progression [78].

Lipids play crucial roles in neuronal structure and physiological functions and are essential for the development and maintenance of the central nervous system [79]. Growing evidence indicates that lipid metabolism disorders are closely associated with neurodegenerative diseases [80]. Abnormal intracellular lipid accumulation induces mitochondrial dysfunction, reduces mitochondrial quantity, and further promotes lipid accumulation [81]. Studies show that PD risk genes such as GBA, LRRK2, and PARK2 are also linked to lipid metabolism [82]. Lipid accumulation impairs mitochondrial function, while mitochondrial dysfunction, in turn, exacerbates lipid metabolism disturbances [73]. Concurrently, lipid metabolism disorders can lead to impaired neuronal function, lysosomal dysfunction, and autophagic deficits, promoting neuronal death and contributing to various neurological diseases [83].

Research indicates that abnormal amino acid metabolism can disrupt synaptic signaling, leading to excitotoxicity and neuronal death. Additionally, oxidative toxicity induced by amino acid metabolism abnormalities is closely associated with ferroptosis [84]. In PD models, the use of dependent sodium channel blockers reduces glutamatergic hyperactivity and inhibits abnormal glutamate release, suggesting that amino acid metabolism disturbances may contribute to neurotoxicity and oxidative toxicity, ultimately resulting in dopaminergic neuron death [85]. Cysteine, a constituent amino acid of glutathione, is critical for antioxidant defense. Reduced cysteine uptake leads to decreased glutathione (GSH) levels, and low GSH concentrations induce OS, mitochondrial dysfunction, protein oxidative damage, and neurodegeneration, thereby promoting PD [86].

These metabolic pathways interact deeply with core mechanisms such as OS, mitochondrial dysfunction, and abnormal α-syn aggregation, collectively forming the complex metabolic background of PD. This integrated perspective provides new insights for understanding disease pathogenesis and developing novel therapeutic strategies.

### Other

Recent studies have revealed that multiple peripheral-central pathways, including the gut-brain axis, spleen-brain axis, liver-brain axis, and skin-brain axis, collectively constitute a complex pathological network in PD. These axes interact with the central nervous system through distinct molecular mechanisms and play significant roles in the initiation, propagation, and progression of PD.

The role of the gut microbiota-brain axis in PD pathogenesis is increasingly well-defined. PD patients exhibit characteristic gut microbiota dysbiosis. Research indicates that as PD severity increases, Akkermansia levels rise, whereas Faecalibacterium and Roseburia decrease with disease progression [87]. Notably, Faecalibacterium and Roseburia are short-chain fatty acid (SCFA)-producing genera that confer beneficial protective effects on the gut, and their reduction may impact the cognitive function and mood of PD patients [88]. The accumulation of Akkermansia promotes intestinal mucosal barrier damage and gut inflammation, facilitating abnormal α-syn aggregation within the intestine, which can ultimately lead to systemic inflammation [89]. SCFAs are crucial for modulating intestinal epithelial barrier and blood–brain barrier integrity, inflammatory responses, and endocrine signaling, playing a vital role in communication along the microbiota-gut-brain axis. While fecal SCFA levels are decreased in PD patients, their levels in plasma, urine, and saliva are elevated. Reduced intestinal SCFA levels are closely associated with worse cognitive and motor function in PD patients [90].

Furthermore, prior to the onset of motor symptoms, over half of PD patients experience non-motor symptoms such as chronic constipation due to enteric nervous system dysfunction [91]. Constipation is the most common gastrointestinal dysfunction in PD. Increased Akkermansia is closely linked to constipation, while constipation severity correlates significantly with reduced Blautia and Faecalibacterium. Gut microbiota dysbiosis in PD patients may increase intestinal permeability, exposing the enteric plexus to various toxins. The overall gut microbial profile in PD is characterized by decreased SCFAs and elevated lipopolysaccharide (LPS) levels [92]. This leads to impaired microglial and astrocytic function, exacerbating neuroinflammation and increasing blood–brain barrier (BBB) permeability. Additionally, microglial activation promotes abnormal α-syn fibril aggregation, augments OS, and results in neurodegeneration and gastrointestinal dysfunction, thereby accelerating PD progression. Moreover, the gut microbiota can produce microbial amyloid proteins, which not only aggravate inflammatory responses but also accelerate α-syn aggregation in the gut. These pathological proteins can then propagate to the central nervous system via the vagus nerve, exacerbating PD development [93]. The therapeutic implications of gut microbiota dysbiosis in PD extend beyond symptom relief and may involve modulating gut-brain axis mechanisms to delay disease progression. Exploring the deep-seated connections between dysbiosis and PD could thus open new avenues for PD treatment.

Additionally, the spleen is a vital peripheral immune organ in the body, harboring diverse immune cells including macrophages, lymphocytes, and dendritic cells, which collectively form a complex immune microenvironment. Reports indicate that in MPTP-induced PD mouse models, a significant increase in splenic macrophages is observed. The rise in M1 macrophages activates the NF-κB signaling pathway, leading to elevated levels of proinflammatory factors [94]. This suggests that the spleen, particularly its macrophage population, may participate in neuroimmune regulation of Parkinson's disease by bridging peripheral and central inflammation.

Research on the liver-brain axis has uncovered the potential significance of the liver in PD. Evidence indicates that α-syn deposition is observed in the livers of both PD models and patients, showing an age-related distribution pattern [42, 95]. Notably, brain-derived α-syn has been found to propagate to the liver, suggesting bidirectional communication of pathological proteins between organs [42]. These findings indicate that the livers of PD mouse models are affected by α-syn aggregation, and α-syn may propagate from the brain to the liver, subsequently inducing hepatic inflammation [42]. This suggests that the liver may be involved in the pathological process of PD, influencing its progression.

Compared with healthy adults, PD patients exhibit significant α-syn deposition in cutaneous autonomic nerve fiber terminals, with notably higher levels in the scalp and cervical skin than in other sites [96]. Growing evidence suggests that α-syn accumulation in cholinergic and adrenergic skin fibers holds important diagnostic value for idiopathic PD [97]. The pathology of α-syn aggregation and dopaminergic neuron loss in the peripheral skin resembles that in the central nervous system, indicating a strong correlation between cutaneous and CNS pathology [98].

These peripheral-central axes provide multiple points of origin for α-syn propagation through mechanisms such as immune activation, pathological protein deposition, and inflammatory signaling, thereby systemically exacerbating central nervous system damage. This systemic perspective opens new pathways for PD prevention and treatment, suggesting that targeting gut microbiota, modulating splenic immunity, clearing hepatic α-syn, or utilizing skin biopsies for early diagnosis may become important future therapeutic strategies.

## Parkinson's disease medications

**Fig. 2:**
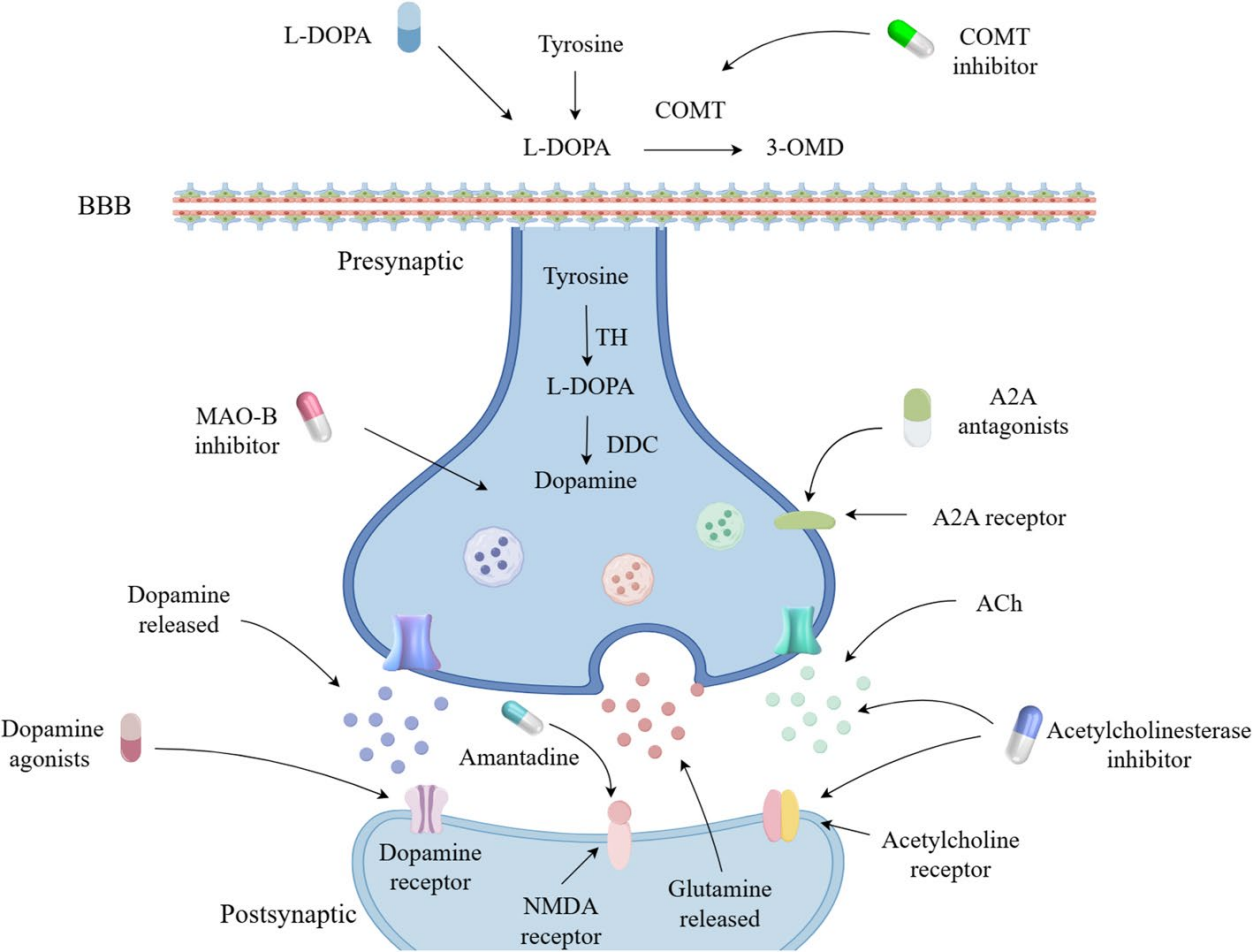
The action sites of different anti-PD drugs. This diagram describes the mechanism of action and the release of neurotransmitters of various anti-PD drugs. BBB = blood–brain barrier; TH = Tyrosine hydroxylase; 3-OMD = 3-O-Methyldopa; DDC = DOPA decarboxylase; MAO-B = Monoamine oxidase B; COMT = Catechol-Omethyl transferase; NMDA = N-Methyl-D-aspartic acid

Although many novel therapeutic technologies for PD are under development, current treatment remains primarily focused on symptomatic management aimed at correcting the imbalance of neurotransmitters in the brain. The primary pharmacological target of anti-PD drugs is the dopaminergic system (Fig. 2), with dopamine-mimetic agents and anticholinergic drugs being the most commonly used in clinical practice (Table 1). Currently, L-DOPA remains the most effective medication for PD. However, long-term administration of L-DOPA is associated with the emergence of adverse effects such as motor complications, which has prompted researchers to actively explore new therapeutic approaches and develop novel anti-PD agents to address disease progression.

**Table 1:** Current clinical drugs for the treatment of PD

Drug category	Drugs	target	efficacy
Dopamine precursor drugs	L-DOPA	DR	Improve motor and non-motor symptoms
DAs	Bromocriptine, cabergoline, lisuride, pergolide, piribedil、pramipexole, ropinirole, rotigotine, apomorphine	DR	Improve movement disorders
MAO-B inhibitor	Selegiline, rasagiline, safinamide	MAO-B	Improve motor symptoms
COMT inhibitor	Tocapone, Entacapone, Opicacapone	COMT	Improve the motion fluctuation caused by L-DOPA
Amantadine	Amantadine	NMDAR	Relieve movement disorders, Reduce the off period time
Anticholinergics	Trihexyphenidyl、Benzat ropine	MAChR	Treatment of PD patients with tremor
Adenosine A2A receptor antagonist	istradefylline	Adenosine A2A receptor	Reduce the off period time, improve movement disorders

*DR* Dopamine Receptor, *Das* Dopamine agonists, *MAO-B* Monoamine oxidase B, *COMT* Catechol-O-methyltransferase, *NMDAR* N-methyl-D-aspartate receptor; MAChR = Muscarinic Acetylcholine Receptor

### Existing drugs for clinical use

#### L—DOPA

The primary cause of PD is the deficiency of dopamine (DA) in the nigrostriatal pathway. In PD patients, the imbalance between dopamine and acetylcholine (ACh) levels in the striatum leads to a series of clinical symptoms. Consequently, traditional therapeutic strategies focus on replenishing cerebral dopamine. L-DOPA, as a dopamine precursor, remains the most effective medication for PD [99]. Combined with peripheral dopa decarboxylase inhibitors in fixed-dose formulations, it is converted into dopamine after crossing the blood–brain barrier, effectively compensating for the dopaminergic deficit in the nigrostriatal pathway and significantly improving core motor symptoms such as bradykinesia, rigidity, and tremor [100], long-term use is associated with motor complications including wearing-off phenomena, dyskinesias, and "on–off" fluctuations, as well as peripheral dopaminergic adverse effects such as nausea, vomiting, and hypotension [101, 102]. Despite its well-established efficacy, L-DOPA therapy faces significant limitations. Compared with other medications, while L-DOPA has a rapid onset of action, its long-term use carries a higher risk of complications.

Recent clinical trials aiming to slow disease progression have failed to meet their primary endpoints, suggesting that dopamine replacement alone is insufficient to halt disease advancement. Therefore, key challenges for future research include balancing short-term symptom control with long-term complication risks, developing non-dopaminergic therapies to address non-motor symptoms, and exploring combination drug strategies to achieve synergistic therapeutic effects.

#### Dopamine agonists

Dopamine receptor agonists are one of the commonly used medications for the treatment of PD. Their mechanism of action involves direct stimulation of postsynaptic dopamine receptors in the striatum, contributing to both neuroprotective and reparative effects. These agents demonstrate considerable efficacy in managing PD symptoms. In early-stage PD, they can be used as monotherapy to delay the introduction of L-DOPA, while in advanced stages, their combination with L-DOPA helps mitigate associated motor complications [103].

Currently, clinical use of dopamine agonists has shifted from earlier ergot derivatives to safer non-ergot agents. These include oral formulations such as pramipexole and ropinirole, the transdermal patch rotigotine, and subcutaneous apomorphine for acute "off"-period rescue [104]. In addition to effectively improving motor symptoms and reducing "off" time, some agents—notably pramipexole—have shown benefits for non-motor symptoms such as depression and sleep disturbances [105, 106]. Orthostatic hypotension, dizziness, drowsiness, and hallucinations are common adverse reactions [107, 108].

Although dopamine agonists offer advantages in delaying motor complications, their overall efficacy remains inferior to that of L-DOPA, and they are associated with a higher incidence of behavioral adverse events. These limitations drive ongoing research efforts aimed at developing next-generation agents with improved receptor selectivity profiles to enhance their overall clinical utility.

#### MAO-B inhibitors

Monoamine oxidase (MAO) is an enzyme embedded in the outer mitochondrial membrane that catalyzes the oxidative deamination of monoamine neurotransmitters and tyramine. It exists in two subtypes, MAO‑A and MAO‑B, with MAO‑B being clinically utilized in the treatment of PD [109]. MAO‑B is a key enzyme involved in the catabolism of dopamine. MAO‑B inhibitors primarily work by inhibiting the metabolism of dopamine in the brain, reducing its deamination, thereby increasing both the amount and duration of dopamine action. Additionally, they may prevent the formation of neurotoxins, thereby slowing the progression of neuronal degeneration [110].

In clinical practice, the value of these agents is reflected in two main aspects: as monotherapy in early disease they can delay the need for L-DOPA, and as adjunctive therapy in moderate to advanced stages they effectively reduce "off" time associated with L-DOPA. The development of this drug class has evolved through three generations. The first-generation agent selegiline, while effective in improving symptoms, has relatively low selectivity, often causes side effects such as insomnia, and requires attention to drug interactions [111, 112]. The second-generation agent rasagiline exhibits higher selectivity and bioavailability, has a milder side-effect profile, does not require dietary restrictions, and may offer potential benefits in slowing disease progression alongside improving motor symptoms [113]. The third-generation agent safinamide achieves high selectivity and reversible inhibition, featuring a unique dual mechanism of action [114]. It not only inhibits MAO‑B but also modulates glutamate release, significantly reducing the incidence of motor fluctuations [115, 116].

Although MAO‑B inhibitors are generally well tolerated, their clinical application still faces key challenges. Future research should focus on developing new compounds with enhanced selectivity and clearly demonstrated disease-modifying effects, as well as exploring their potential for intervention in the prodromal phase of PD.

#### COMT inhibitors

COMT inhibitors primarily function by inhibiting COMT, thereby increasing the concentration of L-DOPA in the brain and preventing its peripheral degradation into 3-O-methyldopa (3-OMD). This effectively prolongs the plasma half-life and duration of action of L-DOPA, which plays a crucial role in the treatment of PD [117]. By protecting L-DOPA from metabolism by peripheral COMT enzymes, COMT inhibitors are important therapeutics for managing motor fluctuations induced by long-term L-DOPA therapy. They improve motor fluctuations through extending central dopamine activity [118]. The core clinical value of this drug class lies in their use as adjuncts to L-DOPA, significantly prolonging patients' "on" time, shortening "off" time, and potentially reducing the total daily dose of L-DOPA.

Currently, the main agents used clinically include tolcapone, entacapone, and opicapone. Among them, tolcapone, the first COMT inhibitor, demonstrates notable efficacy due to its combined peripheral and central action [119]. However, its potential hepatotoxicity risk necessitates strict liver function monitoring, limiting its use as a first-line agent [120]. Entacapone, the most commonly used peripherally selective inhibitor, carries no hepatotoxicity risk but must be administered concomitantly with each L-DOPA dose [121]. Its common side effects include dyskinesia and urine discoloration [122]. The newest generation agent, opicapone, is also a peripherally selective inhibitor. It achieves once-daily dosing owing to its prolonged enzyme inhibitory capacity [123, 124], greatly improving medication convenience, though it is associated with a relatively higher incidence of dyskinesia [125].

Overall, the application of COMT inhibitors requires balancing the clinical benefit of extended "on" time against the risk of exacerbating dyskinesia. Future research should focus on optimizing dosing strategies, clarifying the mechanisms underlying individual differences in treatment response, and exploring their potential in combination with novel therapies, aiming to provide better management strategies for motor fluctuations.

#### Amantadine

Amantadine is a noncompetitive antagonist of the glutamate receptor N-methyl-D-aspartate receptor (NMDAR), exerting both direct and indirect effects on glutamatergic and dopaminergic signaling. By inhibiting NMDAR, it can control dyskinesia and is used in the treatment of L-DOPA-induced dyskinesia [126]. Amantadine possesses not only antiglutamatergic but also anticholinergic properties. It promotes the release of dopamine by stimulating the terminals of nigrostriatal dopaminergic neurons and reduces neuronal reuptake of dopamine, thereby increasing dopamine levels and exerting anti-parkinsonian effects [127].

Its therapeutic value is primarily demonstrated in two aspects: significantly improving dyskinetic symptoms, and effectively prolonging "on" time while shortening "off" time. This makes it the only drug currently approved by the FDA for treating both types of motor complications [128]. Regarding safety, amantadine is generally well-tolerated, with common adverse reactions including hallucinations, dizziness, and constipation, most of which are mild to moderate [129, 130].

However, important limitations remain in its clinical application. Its exact mechanism of action has not been fully elucidated, and its efficacy is primarily evident at the level of symptom control, lacking evidence for disease-modifying effects. Future research needs to further explore its neuromodulatory mechanisms and develop synergistic application strategies with other treatments to better realize its unique value in PD management.

#### Anticholinergics

Anticholinergic drugs were the first class of medications used clinically for the treatment of PD. Trihexyphenidyl and Benztropine are commonly used anticholinergic agents in clinical practice. Their mechanism of action involves blocking cholinergic receptors, thereby reducing the effect of acetylcholine in the nigrostriatal pathway to restore the balance between dopamine and cholinergic neurotransmitters, which underlies their therapeutic effect [131]. Clinically, anticholinergic drugs are often used in combination with L-DOPA or dopamine receptor agonists, primarily for the treatment of PD patients with tremor [132].

The antagonism of acetylcholine can lead to adverse reactions such as cognitive impairment, confusion, blurred vision, hallucinations, urinary retention, and constipation. Cognitive impairment can severely affect patients' quality of life and is also a significant factor contributing to increased mortality in PD patients [133]. Therefore, anticholinergic drugs are generally recommended only for younger patients without cognitive impairment who have poorly controlled tremor [134].

Due to the numerous safety concerns associated with anticholinergic drugs, their clinical application is significantly limited. Future research should focus on elucidating the precise mechanisms underlying PD tremor and developing novel therapeutic strategies with regional specificity that can effectively control tremor while avoiding systemic cholinergic side effects.

#### Adenosine A2A receptor antagonist

In recent years, adenosine A2A receptor antagonists, as non-dopaminergic compounds capable of modulating striatopallidal output to improve motor disability, have been extensively studied [135]. In 2019, the US FDA approved the adenosine A2A receptor antagonist istradefylline as an adjunctive treatment to L-DOPA/carbidopa in adult PD patients experiencing OFF episodes or the wearing-off phenomenon.

Its mechanism of action involves blocking A2A receptors within the indirect pathway of the basal ganglia, thereby modulating the function of motor circuits [136]. This agent is well-tolerated and easy to use, effectively reducing patients' OFF time without significantly exacerbating dyskinesia. Common adverse reactions observed clinically include dyskinesia, somnolence, orthostatic hypotension, nausea, and hallucinations; however, its overall tolerability profile remains favorable [137, 138]. Future research should explore combination strategies of this drug with other therapies to maximize its therapeutic value.

### Emerging treatments

#### Inhaled Levodopa (CVT-301)

To address the issue of OFF episodes in PD patients despite oral L-DOPA administration, an inhaled L-DOPA formulation, CVT-301, has been developed. Utilizing dry powder technology delivered via a breath-actuated inhaler, it bypasses gastrointestinal degradation of L-DOPA. The drug is rapidly absorbed through the alveolar-capillary network and enters the central nervous system, leading to a quicker onset of action [139]. Compared to oral administration, it avoids impaired drug absorption associated with the gastrointestinal tract, thereby rapidly elevating plasma L-DOPA concentrations [140].

Studies have shown that inhalation of CVT-301 significantly increases ON time compared to placebo. Furthermore, it not only effectively reduces the frequency of OFF episodes and improves motor symptoms but also demonstrates a favorable safety and tolerability profile, with no serious adverse events reported. Common adverse reactions include nausea, cough, sputum discoloration, throat irritation, and other respiratory symptoms [141]. As the inhaled L-DOPA is delivered intranasally to the lungs and enters the systemic circulation, it carries a risk of bronchospasm and is not suitable for patients with chronic lung diseases [142].

In terms of clinical application, its mechanism of action defines it as an important tool for symptom management rather than a disease-modifying therapy. As an on-demand treatment, its duration of effect is limited, and it cannot replace long-term strategies such as L-DOPA-carbidopa intestinal gel or continuous subcutaneous apomorphine infusion, which aim to provide stable plasma drug concentrations. Overall, this formulation offers a unique and clinically implemented new option for the symptomatic management of PD, filling a therapeutic gap for rapid rescue of OFF episodes. However, its optimal usage strategy and long-term benefits still require further clarification in broader real-world settings.

#### Levodopa–carbidopa intestinal gel

To improve the utilization of L-DOPA, address its short half-life, and alleviate patients' motor symptoms, several different L-DOPA formulations have been developed. L-DOPA-Carbidopa Intestinal Gel (LCIG) is primarily administered via a portable infusion pump connected to a percutaneous endoscopic gastrojejunostomy (PEG-J) tube. This system delivers the drug directly into the jejunum, bypassing the stomach to prevent interference with absorption, thereby enhancing drug bioavailability and increasing dopamine levels in the body [143].

Studies have shown that after treatment with LCIG in advanced PD patients, both motor and non-motor symptoms were significantly improved, with concurrent enhancements in mood and sleep quality [144]. Multiple other studies have confirmed the efficacy of long-term LCIG use in PD patients. LCIG not only improves dyskinesia and sleep issues but also reduces OFF time and increases ON time [145]. It alleviates motor fluctuations without increasing troublesome dyskinesia. Significant improvements have been observed in PD patients' Unified Dyskinesia Rating Scale and Non-Motor Symptoms Scale scores, thereby enhancing patients' quality of life and reducing caregiver burden [146, 147].

However, as a highly invasive therapy, its clinical application necessitates a comprehensive risk–benefit evaluation. Adverse events are primarily associated with the PEG-J tube, with the most common complications being tube dislocation and stoma site infection [148]. Consequently, although LCIG represents a crucial therapeutic option for advanced PD, its widespread clinical use heavily depends on careful patient selection, management by specialized teams, and a holistic consideration of efficacy, safety, and cost-effectiveness.

#### Subcutaneous apomorphine infusion

Apomorphine is a dopamine agonist with affinity for various dopamine receptor subtypes, characterized by rapid absorption and a short half-life. To address the limitations of oral medications, continuous subcutaneous apomorphine infusion (CSAI) has been developed as a treatment for PD, primarily administered via an assistive device [149]. Studies have demonstrated that PD patients treated with CSAI exhibit a significant reduction in motor fluctuations, and favorable effects are also observed in patients with impulse control disorders [150]. Compared to placebo, CSAI markedly reduces daily OFF time, improves both motor and non-motor symptoms, and demonstrates a favorable safety and tolerability profile. Further research indicates that CSAI is particularly effective for patients with motor fluctuations and severe insomnia [151]. Treatment with CSAI effectively alleviates dyskinesia in PD patients while also significantly improving their non-motor symptoms [152]. Additionally, it can reduce the daily dosage of L-DOPA, thereby minimizing motor complications. As apomorphine is administered subcutaneously, common adverse reactions include local symptoms such as subcutaneous nodules and erythema.

### Potential therapies

With the deepening understanding of the pathological mechanisms underlying PD, current therapeutic strategies have shifted from purely dopaminergic replacement toward disease-modifying interventions targeting core pathological processes. This section focuses on disease-modifying strategies based on non-dopaminergic mechanisms, encompassing multiple directions such as regulation of protein homeostasis, restoration of brain iron metabolism balance, suppression of neuroinflammation, and improvement of energy metabolism. These potential therapies exert specific effects by targeting abnormal α-syn aggregation, correcting brain iron dyshomeostasis, modulating cellular signaling pathways, among other mechanisms, and have demonstrated significant neuroprotective effects in preclinical studies (Table 2). Some candidates have progressed into clinical trials and show promising therapeutic potential, marking a transition toward multitarget disease-modifying approaches in PD treatment and offering new hope for patients.

**Table 2:** Research progress of potential therapeutic drugs for PD

Drugs	Trial number	Development stage	Effect
α-syn misfolding inhibitor			Inhibition of α-syn misfolding
Minzasolmin (UCB0599)	NCT04658186	Phase Ⅱ(2020)	
c-Abl inhibitor			inhibition of c-Abl, inhibition of neurodegeneration
Nilotinib	NCT02954978	Phase Ⅱ (2017)	
Vodobatinib (K0706)	NCT03655236	Phase Ⅱ (2019)	
Risvodetinib (IkT-148009)	NCT05424276	Phase Ⅱ (2023)	
GLP-1 receptor agonists			Clear pathological α-syn, reduce neuroinflammation and movement disorders
Exenatide	NCT04232969	Phase Ⅲ (2020)	
Liraglutide	NCT02953665	Phase Ⅱ (2017)	
Semaglutide	NCT03659682	Phase Ⅱ (2019)	
Lixisenatide	NCT03439943	Phase Ⅱ (2018)	
Statin			Reduce the accumulation of α-syn and reduce neuroinflammation
Lovastatin	NCT03242499	Phase Ⅱ (2017)	
Simvastatin	NCT02787590	Phase Ⅱ (2016)	
Iron chelators			Reduce iron deposition in the substantia nigra, improve motor symptoms
Deferiprone	NCT02655315	Phase Ⅱ (2016)	
Others			
Ambroxol	NCT02914366	Phase Ⅱ(2016)	Increase the activity of GCase, decrease the level of α-syn
Terazosin		—	Alleviate motor symptoms, reduce the risk of PD dementia
Ginsenoside Rg1		—	Reduce neuroinflammation
Melatonin		—	Improve sleep disorders and motor defects

Research progress of potential therapeutic drugs for PD. This table summarizes ongoing and recently completed clinical trials of potential disease-modifying therapies for PD. Data were collected from ClinicalTrials.gov. Only interventional studies (Phase I–III) with available identifiers were included. Search Keywords: PD, Parkinsonism, Lewy body dementia, drug therapy, medication

#### α-Synuclein misfolding inhibitors

The misfolding of α-syn plays a critical role in the pathogenesis of PD. Accordingly, therapeutic strategies targeting this mechanism have led to the development of α-syn misfolding inhibitors such as NPT200-11. Minzasolmin (UCB0599), a single enantiomer of NPT200-11, is an orally bioavailable small molecule that inhibits the early stages of α-syn misfolding, thereby preventing pathological α-syn aggregation [153].

Studies have demonstrated that minzasolmin exhibits substantial blood–brain barrier penetration, rapidly distributing throughout the brain following oral administration [154]. Research in PD animal models has shown that NPT200-11 reduces abnormal α-syn accumulation, suppresses astrocytic activation—thus attenuating neuroinflammation—and normalizes striatal dopamine transporter (DAT) levels, alleviating motor deficits in PD mice [155]. Phase I clinical trials of UCB0599 further confirmed its ability to cross the blood–brain barrier rapidly and distribute into cerebrospinal fluid, with predominant localization in white and gray matter. Notably, food intake did not significantly affect its pharmacokinetics, and the compound demonstrated a favorable safety and tolerability profile [156]. Currently, Phase II clinical studies of UCB0599 are underway.

Its disease-modifying potential lies in the early intervention of the α-syn aggregation cascade, distinguishing it from conventional symptomatic treatments. However, successful clinical development still faces key challenges, including the need to determine optimal dosing and treatment duration, identify validated biomarkers to accurately assess its pathological modifying effects, and confirm long-term safety and efficacy in larger-scale clinical trials.

#### c-Abl inhibitors

c-Abl, a critical non-receptor tyrosine kinase, functions as a key sensor of cellular stress and is widely expressed throughout the brain, playing an indispensable role in neuronal development and survival. Under normal physiological conditions, c-Abl remains in a relatively quiescent state; however, it becomes activated during neurodegeneration [157]. Elevated levels of activated c-Abl have been observed in brain tissues of patients with PD, suggesting that c-Abl inhibitors may hold therapeutic potential for PD. Nilotinib, an oral c-Abl inhibitor initially approved by the FDA for the treatment of chronic myeloid leukemia (CML), has recently been investigated for PD. Studies indicate that nilotinib exhibits favorable safety and tolerability, degrades α-syn, prevents parkin inactivation, and inhibits c-Abl, thereby potentially mitigating neurodegeneration [158, 159]. Despite its acceptable safety profile, nilotinib demonstrated limited clinical benefit in PD patients, likely due to its poor brain penetration and insufficient cerebrospinal fluid concentrations to achieve meaningful c-Abl inhibition.

Building on the lessons learned from nilotinib's shortcomings, new c-Abl inhibitors with improved central nervous system penetration have been developed. Research indicates that vodobatinib and IKT-148009, two next-generation c-Abl inhibitors, exhibit superior brain penetration and more potent inhibition of cerebral c-Abl compared to nilotinib, showing considerable promise for PD treatment [160–162]. Both compounds are currently in Phase II clinical trials, offering the potential to overcome the therapeutic limitations observed with nilotinib. The field of c-Abl inhibition has thus progressed from the clinical setbacks of nilotinib towards a new generation of high-potency agents. Future efforts must focus on core translational objectives, including direct comparative efficacy analyzes and accelerated biomarker development, to overcome existing bottlenecks and successfully advance this therapeutic class from clinical trials to practical clinical application.

#### GLP-1 receptor agonists

Glucagon-like peptide 1 (GLP-1) is a peptide hormone and growth factor that can cross the blood–brain barrier and is involved in the regulation of various physiological functions. Its neuroprotective effects have been demonstrated in preclinical studies. Glucagon-like peptide 1 receptor agonists (GLP-1 RAs) have been widely used clinically for the treatment of type 2 diabetes [163].

Recent studies have shown that GLP-1 RAs can improve synaptic transmission in the striatum and protect neurons from PD-related stressors [164], suggesting the potential therapeutic role of GLP-1 in neurodegenerative diseases. Research indicates that the GLP-1 receptor agonist exenatide (Exendin-4) exhibits significant anti-inflammatory effects in neurodegenerative diseases, can clear pathological α-syn through autophagy, and alleviates motor deficits induced by PD [165, 166]. Compared with several other GLP-1 receptor agonists, exenatide demonstrates stronger penetration of the blood–brain barrier and a higher proportion entering the brain, and its clinical trials are more extensive [167]. Currently, phase III clinical trials on the efficacy of exenatide for PD treatment are underway. Other GLP-1 receptor agonists with potential for treating neurodegenerative diseases, such as liraglutide, semaglutide, and lixisenatide, are in phase II clinical trials.

Although GLP-1 RAs represent a promising candidate direction for PD treatment with an optimistic outlook, they still face research bottlenecks such as unclear mechanisms underlying individual differences in efficacy, challenges in medication adherence, and a lack of long-term safety data. Their successful translation ultimately depends on in-depth analysis of their mechanisms of action, the establishment of precise treatment strategies, and comprehensive evaluation of efficacy, safety, and cost-effectiveness.

#### Statins

Statins are currently widely used in clinical practice as lipid-lowering agents and drugs for the treatment of cardiovascular diseases. Recent studies have shown that lipophilic statins may have certain therapeutic effects on neurodegenerative diseases, potentially exerting their benefits through various mechanisms such as anti-inflammatory and antioxidant actions. Statins are divided into lipophilic and hydrophilic categories. Existing research indicates that long-term use of lipophilic statins can reduce the incidence of PD [168], likely by inhibiting microglial activation and decreasing the accumulation of inflammatory mediators and α-syn, thereby attenuating neuroinflammation [169].

A recent study on lovastatin for PD treatment demonstrated that lovastatin has stronger blood–brain barrier penetration compared to other statins, shows potential therapeutic effects on motor symptoms in early-stage PD patients, and exhibits good tolerability [170]. Additionally, simvastatin is also highly lipophilic and can cross the blood–brain barrier. Studies in PD animal models have shown that simvastatin exerts significant antioxidant and neuroprotective effects against MPP⁺-induced neurotoxicity [171]. However, a recent clinical trial involving patients with moderate PD indicated that simvastatin failed to demonstrate significant therapeutic efficacy, which contrasts sharply with the optimistic results from prior observational studies and animal experiments [172]. This inconsistency in efficacy assessment may stem from differences in disease stage, statin type, and treatment timing, highlighting the uncertainty regarding its clinical benefits. Therefore, larger-scale studies are needed to explore the therapeutic role of statins in PD. Currently, both simvastatin and lovastatin are in phase II clinical trials, and their results will provide higher-level evidence for clarifying the true value of statins in the treatment of PD.

#### Iron chelators

Iron plays a crucial role in the human body; however, studies have revealed that iron levels are significantly elevated in the substantia nigra of PD patients. Excessive iron accumulation can damage the nigrostriatal dopaminergic system, induce OS, and promote the aggregation of misfolded α-syn, thereby accelerating PD progression [173]. Growing evidence suggests that iron chelators may have therapeutic potential in PD. Initially used to treat systemic iron overload, iron chelators have subsequently been found to exert neuroprotective effects and cross the blood–brain barrier [174, 175].

In PD treatment, iron chelators primarily act by reducing abnormal cerebral iron deposition. Their core mechanisms include attenuating iron-catalyzed OS and lipid peroxidation, inhibiting ferroptosis—an iron-dependent form of cell death—and disrupting the toxic coupling between iron and α-syn, thereby mitigating its pathological aggregation [26]. A prior clinical study demonstrated that iron chelation reduced iron deposition in the substantia nigra and improved motor symptoms in PD patients, indicating favorable outcomes [176].

However, a recently published phase II clinical trial using the iron chelator deferiprone reported that deferiprone was ineffective in newly diagnosed PD patients who had not previously received dopaminergic therapy and even worsened both motor and non-motor symptoms [177]. This trial had certain limitations, and the efficacy of deferiprone in patients already on dopaminergic medications requires further validation. Notably, the contradiction between the long-term biosafety profile and the clinical translational potential of iron chelators cannot be overlooked. Animal data and clinical studies indicate that prolonged use may lead to biosafety concerns such as hematological toxicity and depletion of essential trace elements [176, 178].

Therefore, before considering iron chelators as disease-modifying therapies for neurodegenerative disorders requiring lifelong treatment, longer-term clinical studies are necessary to establish systematic safety monitoring protocols and to carefully weigh the potential benefits of reducing cerebral iron load against the risks associated with chronic administration. Moving forward, efforts should focus on developing novel iron chelators with higher brain selectivity and improved safety profiles to achieve an optimal balance between therapeutic efficacy and risk management.

#### Other investigational agents

β-Glucocerebrosidase (GCase) is a lysosomal enzyme encoded by the GBA1 gene. Mutations in GBA1 represent one of the most common genetic risk factors for PD, leading to significantly reduced GCase protein activity in cholinergic neurons and impairing α-syn metabolism, thereby elevating α-synuclein levels in these neurons [179]. Ambroxol, a cough suppressant widely used in clinical practice, has recently been identified as a molecular chaperone that stabilizes and enhances β-glucocerebrosidase activity, offering a unique potential strategy for disease-modifying therapy in PD. The first clinical trial of ambroxol in PD demonstrated its ability to cross into the cerebrospinal fluid and increase glucocerebrosidase protein levels regardless of the presence of GBA1 mutations [180]. Studies have further confirmed that ambroxol can elevate GCase enzymatic activity and reduce α-synuclein levels [181]. It may modulate α-synuclein levels through multiple mechanisms, indicating that the GCase pathway represents a potential therapeutic target for PD. Currently, a phase II clinical trial of ambroxol for PD is underway. Although early results are promising, they must be interpreted with balance, as future findings may reveal limitations in its efficacy. From a translational perspective, ambroxol offers notable advantages due to its established safety profile and low cost, which could facilitate its rapid repurposing for clinical use.

Terazosin (TZ) is an α1-adrenergic receptor antagonist primarily used clinically for treating benign prostatic hyperplasia. However, recent studies suggest its potential therapeutic value in PD. Given the impaired mitochondrial function and reduced ATP levels in PD patients, TZ enhances the activity of phosphoglycerate kinase 1 (PGK1), thereby stimulating glycolysis and increasing cellular ATP levels [182]. Research in PD cellular and animal models treated with MPTP and 6-OHDA has shown that TZ can restore deficient pyruvate and cerebral dopamine levels, elevate in vivo ATP concentrations, alleviate motor symptoms, and consequently suppress or slow neurodegeneration in PD animal models. Furthermore, significant accumulation of TZ in the brain following administration indicates its ability to cross the blood–brain barrier, highlighting its considerable promise for PD treatment [183]. Additional evidence suggests that TZ use is associated with a reduced risk of developing PD, indicating its potential neuroprotective effects [184, 185]. Another study on TZ and cognitive impairment in PD demonstrated that, compared with tamsulosin—another α1-adrenergic receptor antagonist that does not promote glycolysis—treatment with glycolysis-enhancing TZ was associated with a lower risk of dementia in PD patients [186]. The repurposing of TZ offers a novel perspective for PD treatment; however, its precise neuroprotective mechanisms remain incompletely understood. Future rigorous clinical trials are required to clarify its efficacy specificity, address long-term safety concerns in the PD population, and evaluate its potential cost-effectiveness advantages.

Recent studies suggest that ginsenoside Rg1 possesses therapeutic potential for neurodegenerative diseases. In an LPS-induced mouse model of PD, administration of Rg1 was shown to reduce the activation of microglia and astrocytes, decrease levels of pro-inflammatory cytokines such as TNF-α, IL-1β, and IL-6, elevate anti-inflammatory factors, and mitigate the loss of nigral dopaminergic neurons [187, 188]. These findings indicate that Rg1 can attenuate inflammatory responses in LPS-induced PD mice, thereby exerting neuroprotective effects and ameliorating PD-related symptoms. Other studies have demonstrated that treatment with ginsenoside Rg1 in animal models of PD effectively reduces the loss of dopaminergic neurons in the substantia nigra and striatum and alleviates motor deficits [189]. Its protective effects against neurodegenerative diseases are likely mediated through multiple mechanisms, highlighting its considerable promise for future PD therapy. Existing animal data support the favorable long-term safety profile of Rg1, with no significant systemic toxicity, tissue damage, or behavioral abnormalities observed following prolonged administration [190]. Subsequent research should focus on conducting more comprehensive long-term safety evaluations to provide a solid foundation for its eventual clinical translation.

Melatonin is a hormone primarily secreted by the pineal gland, with broad physiological functions in the body. It is mainly involved in regulating circadian rhythms and also possesses anti-inflammatory, antioxidant, and anti-apoptotic properties. Due to its ability to cross the blood–brain barrier, along with its minimal side effects and favorable tolerability, melatonin holds considerable potential for treating sleep disturbances in PD patients [191]. Preclinical studies have demonstrated that melatonin effectively mitigates lipid peroxidation, improves mitochondrial dysfunction, suppresses neuroinflammation, and reduces the loss of nigrostriatal dopaminergic neurons. Its mechanisms involve direct scavenging of free radicals, activation of the Nrf2 pathway to enhance cellular antioxidant defenses, and upregulation of SIRT3 to improve mitochondrial function and biogenesis [192]. Additionally, melatonin inhibits the activation of the NLRP3 inflammasome in microglia, modulates the balance between pro-inflammatory and anti-inflammatory cytokines, and directly interferes with the abnormal aggregation and fibrillation of α-synuclein [193]. In both in vitro and in vivo models of PD, melatonin has been shown to decrease the expression of pro-inflammatory cytokines such as IL-1β and TNF-α while elevating levels of anti-inflammatory factors. Furthermore, melatonin suppresses MPP⁺-induced activation of the NLRP3 inflammasome, thereby preventing neuronal degeneration and microglial activation, and ameliorates motor symptoms in PD mice [194, 195]. Notably, no significant adverse reactions have been reported in patients following melatonin administration, indicating its relative safety and efficacy [196]. Although current research suggests that melatonin may exert multi-target neuroprotective mechanisms beyond sleep improvement, its precise mechanisms of action in PD remain incompletely understood. Future studies should aim to define its optimal therapeutic time window and design biomarker- and pathology-related clinical endpoints to validate its disease-modifying potential.

## Advanced non-pharmacological interventions

Currently, beyond pharmacological treatments, deep brain stimulation (DBS) has become an effective surgical modality for addressing motor complications in PD. This therapy involves implanting electrodes connected to a pulse generator to deliver electrical signals to specific brain targets, thereby modulating neural circuits [197, 198]. Commonly used clinical targets include the subthalamic nucleus (STN) and the globus pallidus internus (GPi), each with distinct therapeutic emphases. STN-DBS demonstrates significant efficacy in improving tremor, rigidity, and bradykinesia, can mildly alleviate anxiety and depression, and enables a substantial reduction in patients' dopaminergic medication requirements [199–201], making it the most widely applied approach in clinical practice. GPi-DBS is particularly effective for medication-induced dyskinesias, effectively ameliorating dystonia and pain, and allows patients to continue dopaminergic medication without an increased risk of dyskinesias [202]. However, DBS is also associated with potential side effects. GPi-DBS may exacerbate dysarthria [203]; STN-DBS, besides the risk of dysarthria, may also cause eyelid opening apraxia [204, 205], and some studies suggest a potential increase in apathy [206]. It is noteworthy that DBS is not suitable for all patients, as its efficacy and safety are highly dependent on precise target localization, individualized parameter programming, and meticulous postoperative management. Therefore, stringent patient selection, surgery performed by an experienced multidisciplinary team, and long-term follow-up are crucial for maximizing therapeutic benefit.

Immunotherapies targeting α-syn aggregates offer new hope for disease-modifying treatment in PD. The Phase II PASADENA study of prasinezumab, a humanized monoclonal antibody targeting the C-terminus, did not meet its primary endpoint [207]. However, exploratory analyzes revealed it could significantly slow the progression of motor symptoms, an effect particularly pronounced in rapid-progressor subgroups, for example, users of monoamine oxidase B inhibitors, patients with diffuse malignant phenotypes [208]. Subsequent research further suggested that its effect on slowing motor and daily functional decline might be sustained long-term [209]. Although its safety profile was generally manageable, infusion reactions were relatively common [210]. These findings indicate that prasinezumab may possess disease-modifying potential in rapidly progressing PD, but its confirmatory efficacy and applicability to broader populations await validation in further clinical trials.

Stem cell therapy, as a highly promising disease-modifying strategy, has achieved key clinical progress in recent years [211]. A landmark Phase I/II clinical trial has been reported. The study involved transplantation of allogeneic iPS-cell-derived dopaminergic progenitors into the putamen of PD patients. Over 24 months of follow-up, the procedure demonstrated a favorable safety and tolerability profile, with no tumor formation or graft-induced dyskinesias observed [212]. ^18^F-DOPA PET imaging confirmed graft survival and dopamine synthesis, with superior effects observed in the high-dose group. Clinical assessments showed improvement in motor symptoms in the majority of patients [213]. This study provides the first evidence for the feasibility of using an off-the-shelf iPS cell product for cell replacement therapy in PD, laying a solid foundation for developing standardized, scalable regenerative medicine therapies and representing a significant step towards curing PD.

Gene-editing therapies aim to correct the genetic defects or pathological processes underlying PD at their source, with the goal of directly intervening in the disease's genetic or molecular etiology, protecting neurons, and halting or delaying disease progression [214]. Research has found that using CRISPR-Cas9 to edit SNCA and reduce α-syn aggregation can improve motor function in mouse models. Additionally, editing the LRRK2 gene has been shown to partially restore dopaminergic neuron function [215, 216]. Gene-editing therapies face future challenges including delivery, safety, and disease heterogeneity, necessitating multidisciplinary strategies to optimize the therapeutic window.

Among existing non-pharmacological treatments, DBS is the current therapy for managing motor complications in mid-to-late-stage patients. Immunotherapy represents an emerging disease treatment strategy in the clinical validation phase, aiming for early intervention to slow pathological progression. Stem cell therapy is a novel approach moving towards the clinic, aimed at repairing damaged neural networks. Gene editing represents a future hope, particularly for genetic forms of PD, aiming to eliminate the disease cause at its origin. They correspond to different stages of PD disease management and different levels of need (Table 3).

**Table 3:** Non-pharmacological interventions for Parkinson's disease

Intervention Type	Mechanism of Action	Clinical Stage	Efficacy
Deep brain stimulation precursor drugs	Electrical stimulation of STN/GPi modulating neural circuits	Widely used in clinical practice	Improves tremor, rigidity, bradykinesia; reduces medication requirements; effectively controls dyskinesia
Immunotherapy	Monoclonal antibody targeting C-terminus of α-synuclein	Phase II clinical trial	Significantly slows motor symptom progression in rapid-progressor subgroups
Stem cell therapy	Transplantation of iPS cell-derived dopaminergic progenitors	Phase I/II clinical trial	Favorable safety and tolerability at 24 months; motor symptoms improved in most patients
Gene editing therapy	CRISPR editing of SNCA/LRRK2 genes	Preclinical research	Improves motor function in animal models

This table summarizes the key non-pharmacological interventions for Parkinson's disease, including deep brain stimulation, immunotherapy, stem cell therapy, and gene editing therapy. For each intervention type, the mechanism of action, current clinical stage, and primary efficacy findings are presented

*STN* Subthalamic nucleus, *GPi* Globus pallidus internus, *iPS* induced pluripotent stem cell

**Fig. 3:**
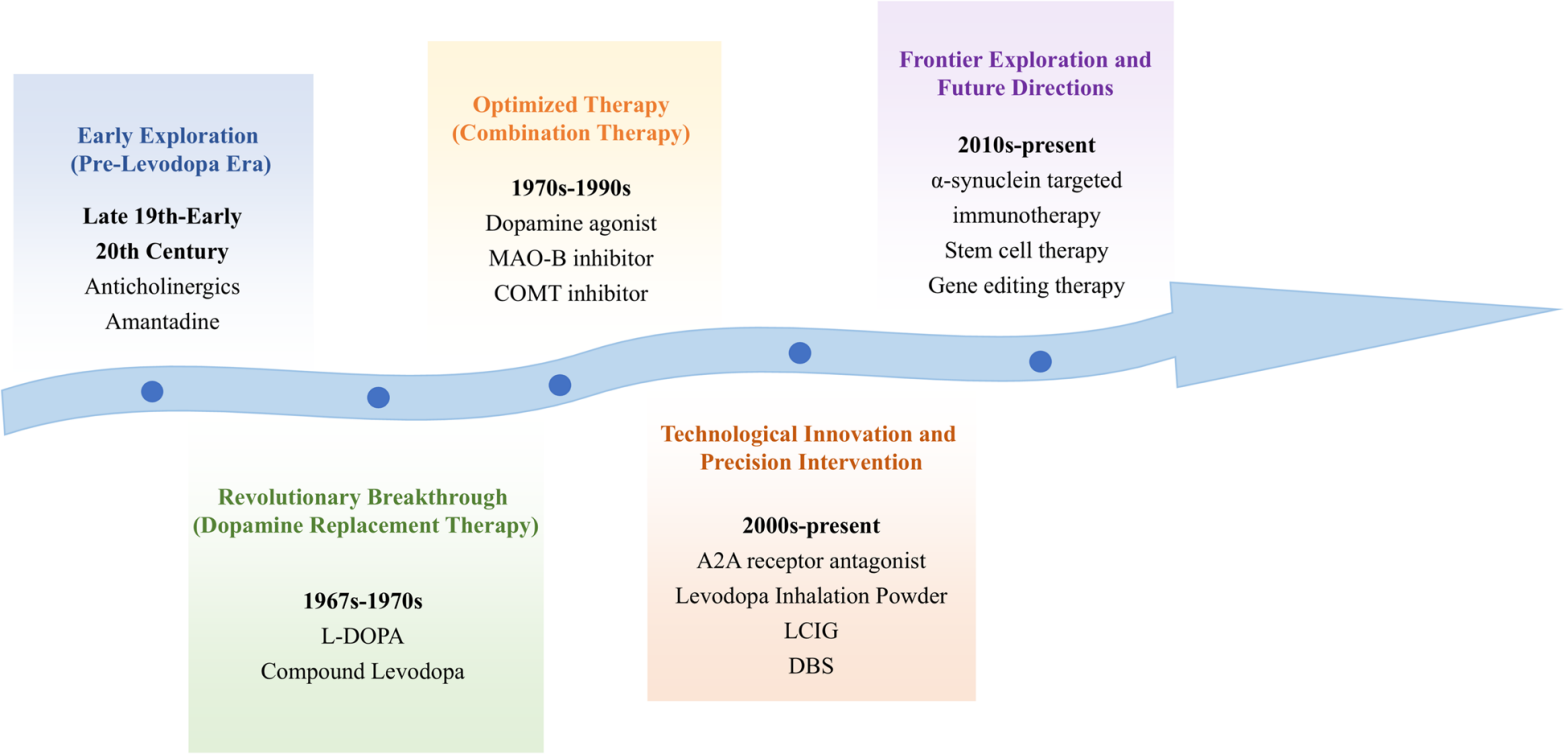
Milestones in the development of Parkinson’s disease therapies. This figure outlines key milestones in the treatment of Parkinson’s disease, whose evolution can be summarized in five phases. The early phase relied on symptomatic management using anticholinergic agents. The 1960s-70 s saw the introduction of L-DOPA and its combination formulations, establishing dopamine replacement as the cornerstone therapy. Subsequently, adjuvant agents such as dopamine agonists, MAO-B inhibitors, and COMT inhibitors, together with continuous infusion strategies, were developed to optimize efficacy and manage motor complications. In the twenty-first century, treatment options expanded further to include novel drug-delivery systems, deep brain stimulation (DBS) surgery, and the first non-dopaminergic agent (adenosine A2A receptor antagonist). Current frontiers are shifting toward disease-modifying strategies, including α-synuclein-targeted immunotherapy, stem cell-based approaches, and gene-editing therapies, marking a transition from symptomatic control to interventions aimed at slowing or repairing the underlying disease process

Currently, pharmacological treatment remains the cornerstone throughout the disease course for controlling core motor symptoms. When medications lead to long-term syndrome complications, DBS intervenes as the most mature non-pharmacological means for effective symptomatic relief. Simultaneously, rehabilitative therapy is an essential component of long-term support. In summary, the treatment of PD has entered an era of integrated management characterized by the synergy between pharmacological and non-pharmacological approaches, equal emphasis on symptomatic and causative treatment, and consideration of both present and future needs. In summary, the evolution of Parkinson's disease treatment can be summarized as follows: a shift from purely symptomatic control to multi-targeted synergistic effects, and from a predominantly drug-based management model to a multi-modal intervention approach (Fig. 3).

## Conclusion

The pathogenesis of PD originates from complex interactions between genetic susceptibility and environmental factors, ultimately converging into four core pathological events: α-syn misfolding, mitochondrial dysfunction, OS, and neuroinflammation. These mechanisms do not operate in isolation but rather form a self-amplifying vicious cycle: mitochondrial damage exacerbates OS and promotes pathological α-syn aggregation; abnormal α-syn in turn impairs mitochondrial function and triggers neuroinflammation; persistent inflammatory responses further aggravate oxidative damage and neuronal loss. Notably, this pathological network is no longer confined to the central nervous system but propagates and amplifies systemically through pathways such as the gut-brain and spleen-brain axes, providing a new perspective for understanding PD as a systemic disorder.

Confronted with the substantial global increase in PD prevalence and the persisting unmet clinical needs in managing both motor and non-motor symptoms, the development of multi-target therapeutic strategies is critically important. The core objective of these strategies is to address the complex, multi-system pathological mechanisms of PD, aiming to achieve superior overall efficacy compared to single-target therapies through synergistic interventions at different pathological nodes. Therefore, in pharmacological management, it is essential not only to employ combination therapies to enhance dopaminergic signaling across multiple pathways and manage motor complications, but also to actively develop novel agents with multi-target profiles and explore treatments targeting non-dopaminergic systems that possess disease-modifying potential. A higher-level multi-target approach involves the deep integration and personalized application of pharmacological and non-pharmacological interventions. For instance, combining optimized post-deep brain stimulation (DBS) medication regimens with targeted rehabilitation can achieve synergy between pharmacological modulation and neural circuit remodeling. This multidimensional strategy aims to concurrently ameliorate motor and non-motor symptoms, balance short-term symptomatic control with long-term disease modification, thereby delaying disease progression and comprehensively improving patients' quality of life. In the future, as the understanding of pathological mechanisms deepens, multi-target therapies are anticipated to evolve toward more precise, dynamic, and individualized paradigms.

To achieve effective treatment for PD, future research must achieve breakthroughs in several key areas. First is the realization of early diagnosis and precise classification of the disease, providing a basis for subsequent targeted, individualized treatment. Second, given the complexity of PD pathogenesis, developing multi-target synergistic therapies capable of simultaneously intervening in multiple critical pathways is particularly crucial. This strategy aims to achieve superior disease control through combined effects. Concurrently, elucidating the interaction mechanisms between the brain and peripheral organs such as the gut and spleen may not only reveal novel pathways for pathological propagation but also uncover a series of entirely new intervention targets. Finally, accelerating the transition of promising frontier strategies—such as gene therapy and cell therapy—from laboratory research to clinical validation and application is a critical step toward achieving the ultimate goals of neural repair and disease modification. Through sustained exploration and systematic integration across these domains, PD treatment paradigms are poised to make a fundamental leap from symptom management toward neural repair and functional restoration.

## Data Availability

Not applicable.

## References

[1] Qi S, Yin P, Wang L, Qu M, Kan GL, Zhang H, et al. Prevalence of parkinson’s disease: a community-based study in China. Mov Disord Off J Mov Disord Soc. 2021;36:2940–4. 10.1002/mds.28762. 10.1002/mds.28762 34390510

[2] GBD 2016 Parkinson’s Disease Collaborators. Global, regional, and national burden of Parkinson’s disease, 1990-2016: a systematic analysis for the Global Burden of Disease Study 2016. Lancet Neurol. 2018;17:939–53. 10.1016/S1474-4422(18)30295-3. 30287051 10.1016/S1474-4422(18)30295-3 PMC6191528

[3] Morris HR, Spillantini MG, Sue CM, Williams-Gray CH. The pathogenesis of parkinson’s disease. Lancet Lond Engl. 2024;403:293–304. 10.1016/S0140-6736(23)01478-2. 10.1016/S0140-6736(23)01478-2 38245249

[4] Global burden of 292 causes of death in 204 countries and territories and 660 subnational locations, 1990–2023: a systematic analysis for the Global Burden of Disease Study 2023. Lancet Lond Engl. 2025;406. 10.1016/S0140-6736(25)01917-8. 10.1016/S0140-6736(25)01917-8 PMC12535838 41092928

[5] Tambasco N, Romoli M, Calabresi P. Levodopa in parkinson’s disease: current status and future developments. Curr Neuropharmacol. 2018;16:1239–52. 10.2174/1570159X15666170510143821. 28494719 10.2174/1570159X15666170510143821 PMC6187751

[6] Li X, Luo X, Cao X, Thomas ER, Wang W, Zhou X, et al. Sirtuins in parkinson’s disease: molecular mechanisms and pathophysiological roles. Ageing Res Rev. 2025;112:102902. 10.1016/j.arr.2025.102902. 41016604 10.1016/j.arr.2025.102902

[7] Liu T, Wu H, Wei J. Beyond the brain: exploring the multi-organ axes in Parkinson’s disease pathogenesis. J Adv Res. 2025;S2090–1232(25):00352–62. 10.1016/j.jare.2025.05.034. 10.1016/j.jare.2025.05.034 PMC12869220 40383292

[8] Mouchaileh N, Cameron J. Device-assisted therapies for Parkinson disease. Aust Prescr. 2025;48:10–7. 10.18773/austprescr.003. 40040736 10.18773/austprescr.2025.003 PMC11875732

[9] Bogers JS, Bloem BR, Den Heijer JM. The etiology of parkinson’s disease: new perspectives from gene-environment interactions. J Park Dis. 2023;13:1281–8. 10.3233/JPD-230250. 10.3233/JPD-230250 PMC10741370 37980685

[10] Coleman C, Martin I. Unraveling parkinson’s disease neurodegeneration: does aging hold the clues? J Park Dis. 2022;12:2321–38. 10.3233/JPD-223363. 10.3233/JPD-223363 PMC9837701 36278358

[11] Ben-Shlomo Y, Darweesh S, Llibre-Guerra J, Marras C, San Luciano M, Tanner C. The epidemiology of parkinson’s disease. Lancet Lond Engl. 2024;403:283–92. 10.1016/S0140-6736(23)01419-8. 10.1016/S0140-6736(23)01419-8 PMC11123577 38245248

[12] Sampson TR, Debelius JW, Thron T, Janssen S, Shastri GG, Ilhan ZE, et al. Gut microbiota regulate motor deficits and neuroinflammation in a model of parkinson’s disease. Cell. 2016;167:1469-1480.e12. 10.1016/j.cell.2016.11.018. 27912057 10.1016/j.cell.2016.11.018 PMC5718049

[13] Westenberger A, Brüggemann N, Klein C. Genetics of parkinson’s disease: from causes to treatment. Cold Spring Harb Perspect Med. 2025;15:a041774. 10.1101/cshperspect.a041774. 39134389 10.1101/cshperspect.a041774 PMC12212002

[14] Polymeropoulos MH, Lavedan C, Leroy E, Ide SE, Dehejia A, Dutra A, et al. Mutation in the alpha-synuclein gene identified in families with Parkinson’s disease. Science. 1997;276:2045–7. 10.1126/science.276.5321.2045. 9197268 10.1126/science.276.5321.2045

[15] Blauwendraat C, Nalls MA, Singleton AB. The genetic architecture of Parkinson’s disease. Lancet Neurol. 2020;19:170–8. 10.1016/S1474-4422(19)30287-X. 31521533 10.1016/S1474-4422(19)30287-X PMC8972299

[16] Cherian A, Divya KP, Vijayaraghavan A. Parkinson’s disease - genetic cause. Curr Opin Neurol. 2023;36:292–301. 10.1097/WCO.0000000000001167. 37366140 10.1097/WCO.0000000000001167

[17] Pedersen CC, Lange J, Førland MGG, Macleod AD, Alves G, Maple-Grødem J. A systematic review of associations between common SNCA variants and clinical heterogeneity in Parkinson’s disease. NPJ Park Dis. 2021;7:54. 10.1038/s41531-021-00196-5. 10.1038/s41531-021-00196-5 PMC8249472 34210990

[18] Rahman AA, Morrison BE. Contributions of VPS35 mutations to Parkinson’s disease. Neuroscience. 2019;401:1–10. 10.1016/j.neuroscience.2019.01.006. 30660673 10.1016/j.neuroscience.2019.01.006 PMC6422337

[19] Jankovic J, Tan EK. Parkinson’s disease: etiopathogenesis and treatment. J Neurol Neurosurg Psychiatry. 2020;91:795–808. 10.1136/jnnp-2019-322338. 32576618 10.1136/jnnp-2019-322338

[20] Wittke C, Petkovic S, Dobricic V, Schaake S, MDS‐endorsed PSP Study Group, Respondek G, et al. Genotype–Phenotype Relations for the Atypical Parkinsonism Genes: MDSGene Systematic Review. Mov Disord. 2021;36:1499–510. 10.1002/mds.28517. 34396589 10.1002/mds.28517 PMC9070562

[21] Chew EG, Liu Z, Li Z, Chung SJ, Lian MM, Tandiono M, et al. Exome sequencing in Asian populations identifies low-frequency and rare coding variation influencing Parkinson’s disease risk. Nat Aging. 2025;5:205–18. 10.1038/s43587-024-00760-7. 39572736 10.1038/s43587-024-00760-7 PMC11839463

[22] Smith L, Schapira AHV. GBA variants and Parkinson disease: mechanisms and treatments. Cells. 2022;11:1261. 10.3390/cells11081261. 35455941 10.3390/cells11081261 PMC9029385

[23] Ye H, Robak LA, Yu M, Cykowski M, Shulman JM. Genetics and pathogenesis of Parkinson’s syndrome. Annu Rev Pathol. 2023;18:95–121. 10.1146/annurev-pathmechdis-031521-034145. 36100231 10.1146/annurev-pathmechdis-031521-034145 PMC10290758

[24] Nalls MA, Blauwendraat C, Vallerga CL, Heilbron K, Bandres-Ciga S, Chang D, et al. Identification of novel risk loci, causal insights, and heritable risk for Parkinson’s disease: a meta-analysis of genome-wide association studies. Lancet Neurol. 2019;18:1091–102. 10.1016/S1474-4422(19)30320-5. 31701892 10.1016/S1474-4422(19)30320-5 PMC8422160

[25] Dorsey ER, De Miranda BR, Hussain S, Bloem BR, Elbaz A, Llibre-Guerra J, et al. Environmental toxicants and Parkinson’s disease: recent evidence, risks, and prevention opportunities. Lancet Neurol. 2025;24:976–86. 10.1016/S1474-4422(25)00287-X. 41109237 10.1016/S1474-4422(25)00287-X

[26] Chen L, Shen Q, Liu Y, Zhang Y, Sun L, Ma X, et al. Homeostasis and metabolism of iron and other metal ions in neurodegenerative diseases. Signal Transduct Target Ther. 2025;10:31. 10.1038/s41392-024-02071-0. 39894843 10.1038/s41392-024-02071-0 PMC11788444

[27] Paul KC, Krolewski RC, Lucumi Moreno E, Blank J, Holton KM, Ahfeldt T, et al. A pesticide and iPSC dopaminergic neuron screen identifies and classifies Parkinson-relevant pesticides. Nat Commun. 2023;14:2803. 10.1038/s41467-023-38215-z. 37193692 10.1038/s41467-023-38215-z PMC10188516

[28] Gao Y, Tang X, Yao J, Sun T, Chen Y, Cheng C, et al. Targeting the bile acid receptor TGR5 with Gentiopicroside to activate Nrf2 antioxidant signaling and mitigate Parkinson’s disease in an MPTP mouse model. J Adv Res. 2025;:S2090–1232(25)00356-X. 10.1016/j.jare.2025.05.039. 10.1016/j.jare.2025.05.039 PMC12869198 40414345

[29] Liang F, Xu Z, Ding L, Zhu Z, Chen M, Shu H, et al. Biomass fuel induces neuroinflammation and neurodegeneration via the astrocyte-microglia IL-17A/IL-17RA pathway. J Hazard Mater. 2025;494:138569. 10.1016/j.jhazmat.2025.138569. 40435626 10.1016/j.jhazmat.2025.138569

[30] Murata H, Barnhill LM, Bronstein JM. Air pollution and the risk of Parkinson’s disease: a review. Mov Disord Off J Mov Disord Soc. 2022;37:894–904. 10.1002/mds.28922. 10.1002/mds.28922 PMC9119911 35043999

[31] Huang M, Bargues-Carot A, Riaz Z, Wickham H, Zenitsky G, Jin H, et al. Impact of environmental risk factors on mitochondrial dysfunction, neuroinflammation, protein misfolding, and oxidative stress in the etiopathogenesis of Parkinson’s disease. Int J Mol Sci. 2022;23:10808. 10.3390/ijms231810808. 36142718 10.3390/ijms231810808 PMC9505762

[32] Domenighetti C, Sugier P-E, Sreelatha AAK, Schulte C, Grover S, Mohamed O, et al. Mendelian randomisation study of smoking, alcohol, and coffee drinking in relation to Parkinson’s disease. J Park Dis. 2022;12:267–82. 10.3233/JPD-212851. 10.3233/JPD-212851 PMC9211765 34633332

[33] Chen C, Wang G-Q, Li D, Zhang F. Microbiota-gut-brain axis in neurodegenerative diseases: molecular mechanisms and therapeutic targets. Mol Biomed. 2025;6:64. 10.1186/s43556-025-00307-1. 40952592 10.1186/s43556-025-00307-1 PMC12436269

[34] Lubomski M, Tan AH, Lim S-Y, Holmes AJ, Davis RL, Sue CM. Parkinson’s disease and the gastrointestinal microbiome. J Neurol. 2020;267:2507–23. 10.1007/s00415-019-09320-1. 31041582 10.1007/s00415-019-09320-1

[35] Zhang W, Ye Y, Song J, Sang T, Xia T, Xie L, et al. Research progress of microbiota-gut-brain axis in Parkinson’s disease. J Integr Neurosci. 2023;22:157. 10.31083/j.jin2206157. 38176929 10.31083/j.jin2206157

[36] Fukasawa N, Tsunoda J, Sunaga S, Kiyohara H, Nakamoto N, Teratani T, et al. The gut-organ axis: clinical aspects and immune mechanisms. Allergol Int Off J Jpn Soc Allergol. 2025;74:197–209. 10.1016/j.alit.2025.01.004. 10.1016/j.alit.2025.01.004 39979198

[37] Claudino Dos Santos JC, Lima MPP, Brito GAdeC, Viana GSdeB. Role of enteric glia and microbiota-gut-brain axis in parkinson disease pathogenesis. Ageing Res Rev. 2023;84:101812. 10.1016/j.arr.2022.101812. 36455790 10.1016/j.arr.2022.101812

[38] Challis C, Hori A, Sampson TR, Yoo BB, Challis RC, Hamilton AM, et al. Gut-seeded α-synuclein fibrils promote gut dysfunction and brain pathology specifically in aged mice. Nat Neurosci. 2020;23:327–36. 10.1038/s41593-020-0589-7. 32066981 10.1038/s41593-020-0589-7 PMC7065967

[39] Ma L, Wang H-B, Hashimoto K. The vagus nerve: an old but new player in brain-body communication. Brain Behav Immun. 2025;124:28–39. 10.1016/j.bbi.2024.11.023. 39566667 10.1016/j.bbi.2024.11.023

[40] Li J, Liu T, Xian M, Zhou K, Wei J. The power of exercise: unlocking the biological mysteries of peripheral-central crosstalk in Parkinson’s disease. J Adv Res. 2025;78:717–32. 10.1016/j.jare.2025.03.001. 40049515 10.1016/j.jare.2025.03.001 PMC12684919

[41] Mok SW-F, Wong VK-W, Lo H-H, de Seabra Rodrigues Dias IR, Leung EL-H, Law BY-K, et al. Natural products-based polypharmacological modulation of the peripheral immune system for the treatment of neuropsychiatric disorders. Pharmacol Ther. 2020;208:107480. 10.1016/j.pharmthera.2020.107480. 31972182 10.1016/j.pharmthera.2020.107480

[42] Reyes JF, Ekmark-Léwen S, Perdiki M, Klingstedt T, Hoffmann A, Wiechec E, et al. Accumulation of alpha-synuclein within the liver, potential role in the clearance of brain pathology associated with Parkinson’s disease. Acta Neuropathol Commun. 2021;9:46. 10.1186/s40478-021-01136-3. 33743820 10.1186/s40478-021-01136-3 PMC7980682

[43] Donadio V, Incensi A, Rizzo G, Furia A, Bonvenga S, Olivola E, et al. Skin intraneural phosphorylated α-synuclein is a highly specific biomarker for early Parkinson’s disease. Brain. 2025. 10.1093/brain/awaf313. 40920014 10.1093/brain/awaf313

[44] Liu X, Yang J, Yuan Y, He Q, Gao Y, Jiang C, et al. Optimization of the detection method for phosphorylated α-synuclein in Parkinson disease by skin biopsy. Front Neurol. 2020;11:569446. 10.3389/fneur.2020.569446. 33101177 10.3389/fneur.2020.569446 PMC7554368

[45] Verma O, Faizan M, Goswami S, Singh MP. An update on the involvement of inflammatory mediators in Parkinson’s disease pathogenesis. Arch Toxicol. 2025;99:3527–52. 10.1007/s00204-025-04088-y. 40461681 10.1007/s00204-025-04088-y

[46] Backman EA, Gardberg M, Luntamo L, Peurla M, Vahlberg T, Borghammer P, et al. Nigral neuroinflammation and dopaminergic neurons in Parkinson’s disease and atypical Parkinsonisms. Ann Neurol. 2025;97:1096–109. 10.1002/ana.27202. 39918108 10.1002/ana.27202

[47] Wang J, Dai L, Chen S, Zhang Z, Fang X, Zhang Z. Protein-protein interactions regulating α-synuclein pathology. Trends Neurosci. 2024;47:209–26. 10.1016/j.tins.2024.01.002. 38355325 10.1016/j.tins.2024.01.002

[48] Moore K, Sengupta U, Puangmalai N, Bhatt N, Kayed R. Polymorphic alpha-synuclein oligomers: characterization and differential detection with novel corresponding antibodies. Mol Neurobiol. 2023;60:2691–705. 10.1007/s12035-023-03211-3. 36707462 10.1007/s12035-023-03211-3 PMC9883140

[49] Day JO, Mullin S. The genetics of Parkinson’s disease and implications for clinical practice. Genes. 2021;12:1006. 10.3390/genes12071006. 34208795 10.3390/genes12071006 PMC8304082

[50] Vázquez-Vélez GE, Zoghbi HY. Parkinson’s disease genetics and pathophysiology. Annu Rev Neurosci. 2021;44:87–108. 10.1146/annurev-neuro-100720-034518. 34236893 10.1146/annurev-neuro-100720-034518

[51] Du X-Y, Xie X-X, Liu R-T. The role of α-Synuclein oligomers in Parkinson’s disease. Int J Mol Sci. 2020;21:8645. 10.3390/ijms21228645. 33212758 10.3390/ijms21228645 PMC7697105

[52] Zhang N, Yan Z, Xin H, Shao S, Xue S, Cespuglio R, et al. Relationship among α-synuclein, aging and inflammation in Parkinson’s disease (Review). Exp Ther Med. 2024;27:23. 10.3892/etm.2023.12311. 38125364 10.3892/etm.2023.12311 PMC10728906

[53] Lv Q-K, Tao K-X, Wang X-B, Yao X-Y, Pang M-Z, Liu J-Y, et al. Role of α-synuclein in microglia: autophagy and phagocytosis balance neuroinflammation in Parkinson’s disease. Inflamm Res. 2023;72:443–62. 10.1007/s00011-022-01676-x. 36598534 10.1007/s00011-022-01676-x

[54] Singh A, Kukreti R, Saso L, Kukreti S. Oxidative stress: a key modulator in neurodegenerative diseases. Mol. 2019;24:1583. 10.3390/molecules24081583. 10.3390/molecules24081583 PMC6514564 31013638

[55] Liu T, Kong X, Qiao J, Wei J. Decoding Parkinson’s disease: the interplay of cell death pathways, oxidative stress, and therapeutic innovations. Redox Biol. 2025;85:103787. 10.1016/j.redox.2025.103787. 40712453 10.1016/j.redox.2025.103787 PMC12312120

[56] Dong-Chen X, Yong C, Yang X, Chen-Yu S, Li-Hua P. Signaling pathways in Parkinson’s disease: molecular mechanisms and therapeutic interventions. Signal Transduct Target Ther. 2023;8:73. 10.1038/s41392-023-01353-3. 36810524 10.1038/s41392-023-01353-3 PMC9944326

[57] Dionísio PA, Amaral JD, Rodrigues CMP. Oxidative stress and regulated cell death in Parkinson’s disease. Ageing Res Rev. 2021;67:101263. 10.1016/j.arr.2021.101263. 33540042 10.1016/j.arr.2021.101263

[58] Tchekalarova J, Tzoneva R. Oxidative stress and aging as risk factors for Alzheimer’s Disease and Parkinson’s disease: the role of the antioxidant melatonin. Int J Mol Sci. 2023;24:3022. 10.3390/ijms24033022. 36769340 10.3390/ijms24033022 PMC9917989

[59] Riaz Z, Richardson GS, Jin H, Zenitsky G, Anantharam V, Kanthasamy A, et al. Nuclear pore and nucleocytoplasmic transport impairment in oxidative stress-induced neurodegeneration: relevance to molecular mechanisms in Pathogenesis of Parkinson’s and other related neurodegenerative diseases. Mol Neurodegener. 2024;19:87. 10.1186/s13024-024-00774-0. 39578912 10.1186/s13024-024-00774-0 PMC11585115

[60] Chen Z, Rasheed M, Deng Y. The epigenetic mechanisms involved in mitochondrial dysfunction: implication for Parkinson’s disease. Brain Pathol. 2022;32:e13012. 10.1111/bpa.13012. 34414627 10.1111/bpa.13012 PMC9048811

[61] Liang J, Su Z, Su G, Rasheed M, Tang S, Sunniya H, et al. Strategies to rescue mitochondria in Parkinson’s disease: the significance of mitochondrial transfer. Ageing Res Rev. 2025;112:102856. 10.1016/j.arr.2025.102856. 40774649 10.1016/j.arr.2025.102856

[62] Henrich MT, Oertel WH, Surmeier DJ, Geibl FF. Mitochondrial dysfunction in Parkinson’s disease - a key disease hallmark with therapeutic potential. Mol Neurodegener. 2023;18:83. 10.1186/s13024-023-00676-7. 37951933 10.1186/s13024-023-00676-7 PMC10640762

[63] Zhang Y-Y, Li X-S, Ren K-D, Peng J, Luo X-J. Restoration of metal homeostasis: a potential strategy against neurodegenerative diseases. Ageing Res Rev. 2023;87:101931. 10.1016/j.arr.2023.101931. 37031723 10.1016/j.arr.2023.101931

[64] Ullah I, Zhao L, Hai Y, Fahim M, Alwayli D, Wang X, et al. Metal elements and pesticides as risk factors for Parkinson’s disease - A review. Toxicol Rep. 2021;8:607–16. 10.1016/j.toxrep.2021.03.009. 33816123 10.1016/j.toxrep.2021.03.009 PMC8010213

[65] Wang B, Fang T, Chen H. Zinc and central nervous system disorders. Nutrients. 2023;15:2140. 10.3390/nu15092140. 37432243 10.3390/nu15092140 PMC10180555

[66] Soman SK, Woodruff MRJ, Dagda RK. The Mitochondrial foundations of Parkinson’s disease: therapeutic implications. Aging Dis. 2025;16:2695–720. 10.14336/AD.2025.0440. 40540721 10.14336/AD.2025.0440 PMC12339092

[67] Kwon HS, Koh S-H. Neuroinflammation in neurodegenerative disorders: the roles of microglia and astrocytes. Transl Neurodegener. 2020;9:42. 10.1186/s40035-020-00221-2. 33239064 10.1186/s40035-020-00221-2 PMC7689983

[68] Gao C, Jiang J, Tan Y, Chen S. Microglia in neurodegenerative diseases: mechanism and potential therapeutic targets. Signal Transduct Target Ther. 2023;8:359. 10.1038/s41392-023-01588-0. 37735487 10.1038/s41392-023-01588-0 PMC10514343

[69] Bido S, Muggeo S, Massimino L, Marzi MJ, Giannelli SG, Melacini E, et al. Microglia-specific overexpression of α-synuclein leads to severe dopaminergic neurodegeneration by phagocytic exhaustion and oxidative toxicity. Nat Commun. 2021;12:6237. 10.1038/s41467-021-26519-x. 34716339 10.1038/s41467-021-26519-x PMC8556263

[70] Xu W, Huang Y, Zhou R. NLRP3 inflammasome in neuroinflammation and central nervous system diseases. Cell Mol Immunol. 2025;22:341–55. 10.1038/s41423-025-01275-w. 40075143 10.1038/s41423-025-01275-w PMC11955557

[71] Heithoff BP, George KK, Phares AN, Zuidhoek IA, Munoz-Ballester C, Robel S. Astrocytes are necessary for blood-brain barrier maintenance in the adult mouse brain. Glia. 2021;69:436–72. 10.1002/glia.23908. 32955153 10.1002/glia.23908 PMC7736206

[72] Wang Q, Yang S, Zhang X, Zhang S, Chen L, Wang W, et al. Inflammasomes in neurodegenerative diseases. Transl Neurodegener. 2024;13:65. 10.1186/s40035-024-00459-0. 39710713 10.1186/s40035-024-00459-0 PMC11665095

[73] Li H, Zeng F, Huang C, Pu Q, Thomas ER, Chen Y, et al. The potential role of glucose metabolism, lipid metabolism, and amino acid metabolism in the treatment of Parkinson’s disease. CNS Neurosci Ther. 2024;30:e14411. 10.1111/cns.14411. 37577934 10.1111/cns.14411 PMC10848100

[74] Komici K, Femminella GD, Bencivenga L, Rengo G, Pagano G. Diabetes mellitus and Parkinson’s disease: a systematic review and meta-analyses. J Park Dis. 2021;11:1585–96. 10.3233/JPD-212725. 10.3233/JPD-212725 34486987

[75] Renaud J, Bassareo V, Beaulieu J, Pinna A, Schlich M, Lavoie C, et al. Dopaminergic neurodegeneration in a rat model of long-term hyperglycemia: preferential degeneration of the nigrostriatal motor pathway. Neurobiol Aging. 2018;69:117–28. 10.1016/j.neurobiolaging.2018.05.010. 29890391 10.1016/j.neurobiolaging.2018.05.010

[76] Pérez-Taboada I, Alberquilla S, Martín ED, Anand R, Vietti-Michelina S, Tebeka NN, et al. Diabetes causes dysfunctional dopamine neurotransmission favoring nigrostriatal degeneration in mice. Mov Disord. 2020;35:1636–48. 10.1002/mds.28124. 32666590 10.1002/mds.28124 PMC7818508

[77] Lv Y-Q, Yuan L, Sun Y, Dou H-W, Su J-H, Hou Z-P, et al. Long-term hyperglycemia aggravates α-synuclein aggregation and dopaminergic neuronal loss in a Parkinson’s disease mouse model. Transl Neurodegener. 2022;11:14. 10.1186/s40035-022-00288-z. 35255986 10.1186/s40035-022-00288-z PMC8900445

[78] Su C-J, Shen Z, Cui R-X, Huang Y, Xu D-L, Zhao F-L, et al. Thioredoxin-interacting protein (TXNIP) regulates Parkin/PINK1-mediated mitophagy in dopaminergic neurons under high-glucose conditions: implications for molecular links between Parkinson’s disease and diabetes. Neurosci Bull. 2020;36:346–58. 10.1007/s12264-019-00459-5. 31939095 10.1007/s12264-019-00459-5 PMC7142185

[79] Ko C-W, Qu J, Black DD, Tso P. Regulation of intestinal lipid metabolism: current concepts and relevance to disease. Nat Rev Gastroenterol Hepatol. 2020;17:169–83. 10.1038/s41575-019-0250-7. 32015520 10.1038/s41575-019-0250-7

[80] Tracey TJ, Kirk SE, Steyn FJ, Ngo ST. The role of lipids in the central nervous system and their pathological implications in amyotrophic lateral sclerosis. Semin Cell Dev Biol. 2021;112:69–81. 10.1016/j.semcdb.2020.08.012. 32962914 10.1016/j.semcdb.2020.08.012

[81] Gao G, Shi Y, Deng H-X, Krainc D. Dysregulation of mitochondrial α-ketoglutarate dehydrogenase leads to elevated lipid peroxidation in CHCHD2-linked Parkinson’s disease models. Nat Commun. 2025;16:1982. 10.1038/s41467-025-57142-9. 40011434 10.1038/s41467-025-57142-9 PMC11865444

[82] F M, D A, G M, G G, C C, I C. Parkinson’s disease-related genes and lipid alteration. Int J Mol Sci. 2021. 10.3390/ijms22147630. 10.3390/ijms22147630 PMC8305702 34299248

[83] M T, C M, Mp A, L C, F Z. Emerging role of HDL in brain cholesterol metabolism and neurodegenerative disorders. Biochim Biophys Acta Mol Cell Biol Lipids. 2022;1867. 10.1016/j.bbalip.2022.159123. 10.1016/j.bbalip.2022.159123 35151900

[84] Iovino L, Tremblay ME, Civiero L. Glutamate-induced excitotoxicity in Parkinson’s disease: the role of glial cells. J Pharmacol Sci. 2020;144:151–64. 10.1016/j.jphs.2020.07.011. 32807662 10.1016/j.jphs.2020.07.011

[85] Pisanò CA, Brugnoli A, Novello S, Caccia C, Keywood C, Melloni E, et al. Safinamide inhibits in vivo glutamate release in a rat model of Parkinson’s disease. Neuropharmacology. 2020;167:108006. 10.1016/j.neuropharm.2020.108006. 32086070 10.1016/j.neuropharm.2020.108006

[86] Dong H, Chen W, Xu K, Zheng L, Wei B, Liu R, et al. High selectivity fluorescence and electrochemical dual-mode detection of glutathione in the serum of Parkinson’s disease model mice and humans. Anal Chem. 2025;97:1318–28. 10.1021/acs.analchem.4c05627. 39783870 10.1021/acs.analchem.4c05627

[87] Zhu M, Liu X, Ye Y, Yan X, Cheng Y, Zhao L, et al. Gut microbiota: a novel therapeutic target for Parkinson’s disease. Front Immunol. 2022;13:937555. 10.3389/fimmu.2022.937555. 35812394 10.3389/fimmu.2022.937555 PMC9263276

[88] Xie A, Ensink E, Li P, Gordevičius J, Marshall LL, George S, et al. Bacterial butyrate in Parkinson’s disease is linked to epigenetic changes and depressive symptoms. Mov Disord Off J Mov Disord Soc. 2022;37:1644–53. 10.1002/mds.29128. 10.1002/mds.29128 PMC9545646 35723531

[89] Kleine Bardenhorst S, Cereda E, Severgnini M, Barichella M, Pezzoli G, Keshavarzian A, et al. Gut microbiota dysbiosis in Parkinson disease: a systematic review and pooled analysis. Eur J Neurol. 2023;30:3581–94. 10.1111/ene.15671. 36593694 10.1111/ene.15671

[90] Chen S-J, Chen C-C, Liao H-Y, Lin Y-T, Wu Y-W, Liou J-M, et al. Association of fecal and plasma levels of short-chain fatty acids with gut microbiota and clinical severity in patients with Parkinson disease. Neurology. 2022;98:e848–58. 10.1212/WNL.0000000000013225. 34996879 10.1212/WNL.0000000000013225 PMC8883514

[91] Salim S, Ahmad F, Banu A, Mohammad F. Gut microbiome and Parkinson’s disease: perspective on pathogenesis and treatment. J Adv Res. 2023;50:83–105. 10.1016/j.jare.2022.10.013. 36332796 10.1016/j.jare.2022.10.013 PMC10403695

[92] Dogra N, Mani RJ, Katare DP. The gut-brain axis: two ways signaling in Parkinson’s disease. Cell Mol Neurobiol. 2022;42:315–32. 10.1007/s10571-021-01066-7. 33649989 10.1007/s10571-021-01066-7 PMC11441216

[93] Sampson TR, Challis C, Jain N, Moiseyenko A, Ladinsky MS, Shastri GG, et al. A gut bacterial amyloid promotes α-synuclein aggregation and motor impairment in mice. Elife. 2020;9:e53111. 10.7554/eLife.53111. 32043464 10.7554/eLife.53111 PMC7012599

[94] Yan A, Zhang Y, Lin J, Song L, Wang X, Liu Z. Partial depletion of peripheral M1 macrophages reverses motor deficits in MPTP-treated mouse by suppressing neuroinflammation and dopaminergic neurodegeneration. Front Aging Neurosci. 2018;10:160. 10.3389/fnagi.2018.00160. 29922149 10.3389/fnagi.2018.00160 PMC5996129

[95] Y C, M M, R Z, C G, X D, J W, et al. Dairy-rich diet triggers hepatic α-synuclein pathology via the liver-brain axis in GBA1-related Parkinson’s disease. NPJ Park Dis. 2025;11. 10.1038/s41531-025-01211-9. 10.1038/s41531-025-01211-9 PMC12748959 41390841

[96] Peng H, Chen S, Wu S, Shi X, Ma J, Yang H, et al. Alpha-synuclein in skin as a high-quality biomarker for Parkinson’s disease. J Neurol Sci. 2023;451:120730. 10.1016/j.jns.2023.120730. 37454572 10.1016/j.jns.2023.120730

[97] Oizumi H, Yamasaki K, Suzuki H, Ohshiro S, Saito Y, Murayama S, et al. Phosphorylated alpha-synuclein in Iba1-positive macrophages in the skin of patients with Parkinson’s disease. Ann Clin Transl Neurol. 2022;9:1136–46. 10.1002/acn3.51610. 35750465 10.1002/acn3.51610 PMC9380156

[98] Han Y, Wu D, Wang Y, Xie J, Zhang Z. Skin alpha-synuclein deposit patterns: a predictor of Parkinson’s disease subtypes. EBioMedicine. 2022;80:104076. 10.1016/j.ebiom.2022.104076. 35644126 10.1016/j.ebiom.2022.104076 PMC9148991

[99] Tanner CM, Ostrem JL. Parkinson’s disease. N Engl J Med. 2024;391:442–52. 10.1056/NEJMra2401857. 39083773 10.1056/NEJMra2401857

[100] Zhang F, Liu M, Tuo J, Zhang L, Zhang J, Yu C, et al. Levodopa-induced dyskinesia: interplay between the N-methyl-D-aspartic acid receptor and neuroinflammation. Front Immunol. 2023;14:1253273. 10.3389/fimmu.2023.1253273. 37860013 10.3389/fimmu.2023.1253273 PMC10582719

[101] Murakami H, Shiraishi T, Umehara T, Omoto S, Iguchi Y. Recent advances in drug therapy for Parkinson’s disease. Intern Med Tokyo Jpn. 2023;62:33–42. 10.2169/internalmedicine.8940-21. 10.2169/internalmedicine.8940-21 PMC9876715 35110492

[102] Bandopadhyay R, Mishra N, Rana R, Kaur G, Ghoneim MM, Alshehri S, et al. Molecular mechanisms and therapeutic strategies for levodopa-induced dyskinesia in Parkinson’s disease: a perspective through preclinical and clinical evidence. Front Pharmacol. 2022;13:805388. 10.3389/fphar.2022.805388. 35462934 10.3389/fphar.2022.805388 PMC9021725

[103] Jing X-Z, Yang H-J, Taximaimaiti R, Wang X-P. Advances in the therapeutic use of non-ergot dopamine agonists in the treatment of motor and non-motor symptoms of Parkinson’s disease. Curr Neuropharmacol. 2023;21:1224–40. 10.2174/1570159X20666220915091022. 36111769 10.2174/1570159X20666220915091022 PMC10286583

[104] Ferraiolo M, Hermans E. The complex molecular pharmacology of the dopamine D2 receptor: implications for pramipexole, ropinirole, and rotigotine. Pharmacol Ther. 2023;245:108392. 10.1016/j.pharmthera.2023.108392. 36958527 10.1016/j.pharmthera.2023.108392

[105] Jiang D-Q, Jiang L-L, Wang Y, Li M-X. The role of pramipexole in the treatment of patients with depression and Parkinson’s disease: a meta-analysis of randomized controlled trials. Asian J Psychiatry. 2021;61:102691. 10.1016/j.ajp.2021.102691. 10.1016/j.ajp.2021.102691 33992852

[106] Raeder V, Boura I, Leta V, Jenner P, Reichmann H, Trenkwalder C, et al. Rotigotine transdermal patch for motor and non-motor Parkinson’s Disease: a review of 12 years’ clinical experience. CNS Drugs. 2021;35:215–31. 10.1007/s40263-020-00788-4. 33559846 10.1007/s40263-020-00788-4 PMC7871129

[107] Stocchi F, Rascol O, Poewe W, Chaudhuri KR, Kassubek J, Lopez Manzanares L, et al. Apomorphine sublingual film compared with subcutaneous apomorphine for OFF episodes in Parkinson’s Disease: an open-label, randomized, crossover study. J Park Dis. 2023;13:1329–42. 10.3233/JPD-230072. 10.3233/JPD-230072 PMC10741320 37980683

[108] Chen X-T, Zhang Q, Wen S-Y, Chen F-F, Zhou C-Q. Efficacy and safety of non-ergot dopamine-receptor agonists as an adjunct to levodopa in advanced Parkinson’s disease: a network meta-analysis. Eur J Neurol. 2023;30:762–73. 10.1111/ene.15635. 36380711 10.1111/ene.15635 PMC10099912

[109] Alborghetti M, Bianchini E, De Carolis L, Galli S, Pontieri FE, Rinaldi D. Type-B monoamine oxidase inhibitors in neurological diseases: clinical applications based on preclinical findings. Neural Regen Res. 2024;19:16–21. 10.4103/1673-5374.375299. 37488838 10.4103/1673-5374.375299 PMC10479837

[110] Yy T, P J, Sd C. Monoamine Oxidase-B inhibitors for the treatment of Parkinson’s disease: past, present, and future. J Park Dis. 2022;12. 10.3233/JPD-212976. 10.3233/JPD-212976 PMC8925102 34957948

[111] Wang K, Liu Z-H, Li X-Y, Li Y-F, Li J-R, Hui J-J, et al. Efficacy and safety of selegiline for the treatment of Parkinson’s disease: a systematic review and meta-analysis. Front Aging Neurosci. 2023;15:1134472. 10.3389/fnagi.2023.1134472. 37113570 10.3389/fnagi.2023.1134472 PMC10126343

[112] Jost WH. A critical appraisal of MAO-B inhibitors in the treatment of Parkinson’s disease. J Neural Transm Vienna Austria 1996. 2022;129:723–36. 10.1007/s00702-022-02465-w. 10.1007/s00702-022-02465-w PMC9188534 35107654

[113] Su W, Liang Z, Mao W, Shao M, Hu X, Wu Y, et al. Safety and effectiveness of rasagiline in Chinese patients with Parkinson’s Disease: a prospective, multicenter, non-interventional post-marketing study. Drug Saf. 2023;46:637–46. 10.1007/s40264-023-01288-2. 37195560 10.1007/s40264-023-01288-2 PMC10279558

[114] Wasan H, Singh D, Kh R. Safinamide in neurological disorders and beyond: evidence from preclinical and clinical studies. Brain Res Bull. 2021;168:165–77. 10.1016/j.brainresbull.2020.12.018. 33387637 10.1016/j.brainresbull.2020.12.018

[115] Stocchi F, Antonini A, Berg D, Bergmans B, Jost W, Katzenschlager R, et al. Safinamide in the treatment pathway of Parkinson’s disease: a European Delphi Consensus. NPJ Park Dis. 2022;8:17. 10.1038/s41531-022-00277-z. 10.1038/s41531-022-00277-z PMC8861053 35190544

[116] Abbruzzese G, Barone P, Lopiano L, Stocchi F. The current evidence for the use of safinamide for the treatment of Parkinson’s Disease. Drug Des Devel Ther. 2021;15:2507–17. 10.2147/DDDT.S302673. 34140766 10.2147/DDDT.S302673 PMC8203199

[117] Salamon A, Zádori D, Szpisjak L, Klivényi P, Vécsei L. What is the impact of catechol-O-methyltransferase (COMT) on Parkinson’s disease treatment? Expert Opin Pharmacother. 2022;23:1123–8. 10.1080/14656566.2022.2060738. 35373688 10.1080/14656566.2022.2060738

[118] Regensburger M, Ip CW, Kohl Z, Schrader C, Urban PP, Kassubek J, et al. Clinical benefit of MAO-B and COMT inhibition in Parkinson’s disease: practical considerations. J Neural Transm Vienna Austria 1996. 2023;130:847–61. 10.1007/s00702-023-02623-8. 10.1007/s00702-023-02623-8 PMC10199833 36964457

[119] Artusi CA, Sarro L, Imbalzano G, Fabbri M, Lopiano L. Safety and efficacy of tolcapone in Parkinson’s disease: systematic review. Eur J Clin Pharmacol. 2021;77:817–29. 10.1007/s00228-020-03081-x. 33415500 10.1007/s00228-020-03081-x PMC8128808

[120] Song Z, Zhang J, Xue T, Yang Y, Wu D, Chen Z, et al. Different Catechol-O-Methyl Transferase Inhibitors in Parkinson’s Disease: a Bayesian network meta-analysis. Front Neurol. 2021;12:707723. 10.3389/fneur.2021.707723. 34630283 10.3389/fneur.2021.707723 PMC8497751

[121] Reichmann H. Real-world considerations regarding the use of the combination of levodopa, carbidopa, and entacapone (Stalevo® ) in Parkinson’s disease. Eur J Neurol. 2023;30(2):15–20. 10.1111/ene.15992. 37489705 10.1111/ene.15992

[122] Poewe W. Catechol-O-methyltransferase inhibition with entacapone: evidence from controlled clinical trials in Parkinson’s disease. Eur J Neurol. 2023;30(2):9–14. 10.1111/ene.15993. 37493495 10.1111/ene.15993

[123] Bologna M, Guerra A, Colella D, Birreci D, Costa D, Cannavacciuolo A, et al. Objective assessment of the effects of opicapone in Parkinson’s disease through kinematic analysis. Neurol Sci Off J Ital Neurol Soc Ital Soc Clin Neurophysiol. 2024;45:2035–46. 10.1007/s10072-023-07233-6. 10.1007/s10072-023-07233-6 PMC11021230 38091213

[124] Berger AA, Winnick A, Izygon J, Jacob BM, Kaye JS, Kaye RJ, et al. Opicapone, a novel catechol-o-methyl transferase inhibitor, for treatment of Parkinson’s Disease “Off” Episodes. Health Psychol Res. 2022;10:36074. 10.52965/001c.36074. 35774903 10.52965/001c.36074 PMC9239372

[125] Feldman M, Margolesky J. Opicapone for the treatment of Parkinson’s disease: a review. Int J Neurosci. 2023;133:532–43. 10.1080/00207454.2021.1929217. 33980110 10.1080/00207454.2021.1929217

[126] Mehta SH, Pahwa R, Tanner CM, Hauser RA, Johnson R. Effects of Gocovri (Amantadine) extended release capsules on non-motor symptoms in patients with Parkinson’s disease and dyskinesia. Neurol Ther. 2021;10:307–20. 10.1007/s40120-021-00246-3. 33864229 10.1007/s40120-021-00246-3 PMC8140024

[127] Hauser RA, Mehta SH, Kremens D, Chernick D, Formella AE. Effects of Gocovri (Amantadine) extended-release capsules on motor aspects of experiences of daily living in people with Parkinson’s disease and dyskinesia. Neurol Ther. 2021;10:739–51. 10.1007/s40120-021-00256-1. 34024025 10.1007/s40120-021-00256-1 PMC8571461

[128] Hauser RA, Lytle J, Formella AE, Tanner CM. Amantadine delayed release/extended release capsules significantly reduce OFF time in Parkinson’s disease. NPJ Park Dis. 2022;8:29. 10.1038/s41531-022-00291-1. 10.1038/s41531-022-00291-1 PMC8933492 35304480

[129] An X, Su L, Yang Q, Shen B, Gan L, Jun Ji J, et al. A spatiotemporal hypergraph self-attention neural networks framework for the identification and pharmacological efficacy assessment of Parkinson’s disease motor symptoms. NPJ Park Dis. 2025;11:338. 10.1038/s41531-025-01187-6. 10.1038/s41531-025-01187-6 PMC12658025 41298476

[130] Rujirussawarawong S, Aungsumart S, Kasemsuk C, Limotai N. Efficacy and safety of oral amantadine in Parkinson’s disease with dyskinesia and motor fluctuations: a systematic review and meta-analysis of randomised controlled trials. BMJ Neurol Open. 2025;7:e001115. 10.1136/bmjno-2025-001115. 40756069 10.1136/bmjno-2025-001115 PMC12314832

[131] Goetz CG. Historical perspectives of Parkinson’s disease: early clinical descriptions and neurological therapies. Cold Spring Harb Perspect Med. 2025;15:a041642. 10.1101/cshperspect.a041642. 38858084 10.1101/cshperspect.a041642 PMC11960689

[132] Vanegas-Arroyave N, Caroff SN, Citrome L, Crasta J, McIntyre RS, Meyer JM, et al. An evidence-based update on anticholinergic use for drug-induced movement disorders. CNS Drugs. 2024;38:239–54. 10.1007/s40263-024-01078-z. 38502289 10.1007/s40263-024-01078-z PMC10980662

[133] Malkiewicz JJ, Kasprzyk AG, Waksmundzki D, Węgrzynek J, Chmiela T, Siuda J. Risk factors for dementia in Parkinson’s disease - the overuse of anticholinergic drugs. Neurol Neurochir Pol. 2023;57:405–13. 10.5603/PJNNS.a2023.0041. 37357543 10.5603/PJNNS.a2023.0041

[134] Pirker W, Katzenschlager R, Hallett M, Poewe W. Pharmacological treatment of tremor in parkinson’s disease revisited. J Park Dis. 2023;13:127–44. 10.3233/JPD-225060. 10.3233/JPD-225060 PMC10041452 36847017

[135] Waggan I, Rissanen E, Tuisku J, Joutsa J, Helin S, Parkkola R, et al. Adenosine A2A receptor availability in patients with early- and moderate-stage Parkinson’s disease. J Neurol. 2023;270:300–10. 10.1007/s00415-022-11342-1. 36053386 10.1007/s00415-022-11342-1 PMC9813038

[136] Mori A, Chen J-F, Uchida S, Durlach C, King SM, Jenner P. The pharmacological potential of adenosine A2A receptor antagonists for treating Parkinson’s disease. Mol Basel Switz. 2022;27:2366. 10.3390/molecules27072366. 10.3390/molecules27072366 PMC9000505 35408767

[137] Isaacson SH, Betté S, Pahwa R. Istradefylline for OFF episodes in Parkinson’s disease: a us perspective of common clinical scenarios. Degener Neurol Neuromuscul Dis. 2022;12:97–109. 10.2147/DNND.S245197. 35910426 10.2147/DNND.S245197 PMC9329678

[138] Sako W, Kogo Y, Koebis M, Kita Y, Yamakage H, Ishida T, et al. Comparative efficacy and safety of adjunctive drugs to levodopa for fluctuating Parkinson’s disease - network meta-analysis. NPJ Park Dis. 2023;9:143. 10.1038/s41531-023-00589-8. 10.1038/s41531-023-00589-8 PMC10584871 37853009

[139] Rascol O. CVT-301 for Parkinson’s disease: dose and effect size issues. Lancet Neurol. 2019;18:128–30. 10.1016/S1474-4422(18)30496-4. 30663600 10.1016/S1474-4422(18)30496-4

[140] Lipp MM, Hickey JA, Langer R, LeWitt AP. A technology evaluation of CVT-301 (Inbrija): an inhalable therapy for treatment of Parkinson’s disease. Expert Opin Drug Deliv. 2021;18:1559–69. 10.1080/17425247.2021.1960820. 34311641 10.1080/17425247.2021.1960820

[141] Glenardi G, Handayani T, Barus J, Mangkuliguna G. Inhaled Levodopa (CVT-301) for the treatment of parkinson disease: a systematic review and meta-analysis of randomized controlled trials. Neurol Clin Pract. 2022;12:139–48. 10.1212/CPJ.0000000000001143. 35747892 10.1212/CPJ.0000000000001143 PMC9208397

[142] Jost HW, Kulisevsky J, LeWitt AP. Inhaled levodopa for threatening impending OFF episodes in managing Parkinson’s disease. J Neural Transm Vienna Austria 1996. 2023;130:821–6. 10.1007/s00702-023-02636-3. 10.1007/s00702-023-02636-3 37087697

[143] Koeglsperger T, Berberovic E, Dresel C, Haferkamp S, Kassubek J, Müller R, et al. Real-world experience with continuous subcutaneous foslevodopa/foscarbidopa infusion: insights and recommendations. J Neural Transm. 2025. 10.1007/s00702-025-02911-5. 40121314 10.1007/s00702-025-02911-5 PMC12855247

[144] Chaudhuri RK, Antonini A, Pahwa R, Odin P, Titova N, Thakkar S, et al. Effects of levodopa-carbidopa intestinal gel on dyskinesia and non-motor symptoms including sleep: results from a meta-analysis with 24-month follow-up. J Parkinsons Dis. 2022;12:2071–83. 10.3233/JPD-223295. 35964203 10.3233/JPD-223295 PMC9661331

[145] Lungu M, Oprea DV, Apostol LL, Elkan ME, Ionescu MA, Tudor A, et al. Levodopa-carbidopa-entacapone intestinal gel for advanced Parkinson’s disease-results from a monocentric study evaluating both motor and non-motor manifestations. Biomedicines. 2025;13:2191. 10.3390/biomedicines13092191. 41007754 10.3390/biomedicines13092191 PMC12467503

[146] Nomoto Y, Furihata M, Hagiwara H, Ishino H, Yano S, Okawa H, et al. Transgastric Jejunostomy (PEG-J) for continuous infusion of levodopa-carbidopa intestinal gel: an approach for Parkinson’s disease treatment. Med Sci Monit Int Med J Exp Clin Res. 2023;29:e941285. 10.12659/MSM.941285. 10.12659/MSM.941285 PMC10429380 37571821

[147] Chaudhuri RK, Kovács N, Pontieri EF, Aldred J, Bourgeois P, Davis LT, et al. Levodopa carbidopa intestinal gel in advanced Parkinson’s disease: DUOGLOBE final 3-year results. J Park Dis. 2023;13:769–83. 10.3233/JPD-225105. 10.3233/JPD-225105 PMC10473130 37302039

[148] Vekhande C, Hamed M, Tremain G, Mah J, Shetty A, Lazarescu A, et al. Levodopa-Carbidopa Intestinal Gel for Parkinson’s Disease over 11 years: One Center’s “Real-World” Experience. Can J Neurol Sci J Can Sci Neurol. 2024;51:379–86. 10.1017/cjn.2023.251. 10.1017/cjn.2023.251 37462070

[149] Yu JRT, Kundrick A, Panganiban EC, Sy MA, Anis S, Fernandez HH. Therapeutic innovations for the symptomatic treatment of Parkinson’s disease: focus on technology-based therapies. J Neural Transm Vienna Austria 1996. 2025. 10.1007/s00702-025-02915-1. 10.1007/s00702-025-02915-1 40119890

[150] Meira B, Degos B, Corsetti E, Doulazmi M, Berthelot E, Virbel-Fleischman C, et al. Long-term effect of apomorphine infusion in advanced Parkinson’s disease: a real-life study. NPJ Parkinsons Dis. 2021;7:50. 10.1038/s41531-021-00194-7. 34117268 10.1038/s41531-021-00194-7 PMC8196159

[151] Garon M, Scharfenort M, Antonini A, Odin P, Ljung H. Cognitive outcomes of infusion therapies in Parkinson’s disease: a comprehensive systematic review. Parkinsonism Relat Disord. 2025;138:107950. 10.1016/j.parkreldis.2025.107950. 40731234 10.1016/j.parkreldis.2025.107950

[152] Santos-García D, Solleiro Á, González-Ortega G, Mir P, López-Ariztegui N, Legarda I, et al. Impact of device-aided therapies on quality of life in patients with Parkinson’s disease. A comparative multicenter observational study. J Neural Transm Vienna Austria 1996. 2025. 10.1007/s00702-025-03066-z. 10.1007/s00702-025-03066-z 41251763

[153] Price DL, Khan A, Angers R, Cardenas A, Prato MK, Bani M, et al. In vivo effects of the alpha-synuclein misfolding inhibitor minzasolmin supports clinical development in Parkinson’s disease. NPJ Park Dis. 2023;9:114. 10.1038/s41531-023-00552-7. 10.1038/s41531-023-00552-7 PMC10352257 37460603

[154] Mercier J, Bani M, Colson A-O, Germani M, Lalla M, Plisson C, et al. Evaluation and Application of a PET Tracer in Preclinical and Phase 1 Studies to Determine the Brain Biodistribution of Minzasolmin (UCB0599). Mol Imaging Biol. 2024;26:310–21. 10.1007/s11307-023-01878-7. 38110790 10.1007/s11307-023-01878-7 PMC10973027

[155] Price DL, Koike MA, Khan A, Wrasidlo W, Rockenstein E, Masliah E, et al. The small molecule alpha-synuclein misfolding inhibitor, NPT200-11, produces multiple benefits in an animal model of Parkinson’s disease. Sci Rep. 2018;8:16165. 10.1038/s41598-018-34490-9. 30385782 10.1038/s41598-018-34490-9 PMC6212487

[156] Smit JW, Basile P, Prato MK, Detalle L, Mathy F-X, Schmidt A, et al. Phase 1/1b studies of UCB0599, an oral inhibitor of α-Synuclein misfolding, including a randomized study in Parkinson’s disease. Mov Disord Off J Mov Disord Soc. 2022;37:2045–56. 10.1002/mds.29170. 10.1002/mds.29170 PMC9804489 35959805

[157] Werner MH, Olanow CW. Parkinson’s disease modification through Abl kinase inhibition: an opportunity. Mov Disord Off J Mov Disord Soc. 2022;37:6–15. 10.1002/mds.28858. 10.1002/mds.28858 PMC8770606 34816484

[158] Wu J, Xu X, Zheng L, Mo J, Jin X, Bao Y. Nilotinib inhibits microglia-mediated neuroinflammation to protect against dopaminergic neuronal death in Parkinson’s disease models. Int Immunopharmacol. 2021;99:108025. 10.1016/j.intimp.2021.108025. 34364303 10.1016/j.intimp.2021.108025

[159] Pagan FL, Hebron ML, Wilmarth B, Torres-Yaghi Y, Lawler A, Mundel EE, et al. Nilotinib effects on safety, tolerability, and potential biomarkers in Parkinson disease: a phase 2 randomized clinical trial. JAMA Neurol. 2020;77:309–17. 10.1001/jamaneurol.2019.4200. 31841599 10.1001/jamaneurol.2019.4200 PMC6990742

[160] Walsh RR, Damle NK, Mandhane S, Piccoli SP, Talluri RS, Love D, et al. Plasma and cerebrospinal fluid pharmacokinetics of vodobatinib, a neuroprotective c-Abl tyrosine kinase inhibitor for the treatment of Parkinson’s disease. Parkinsonism Relat Disord. 2023;108:105281. 10.1016/j.parkreldis.2023.105281. 36717298 10.1016/j.parkreldis.2023.105281

[161] Karuppagounder SS, Wang H, Kelly T, Rush R, Nguyen R, Bisen S, et al. The c-Abl inhibitor IkT-148009 suppresses neurodegeneration in mouse models of heritable and sporadic Parkinson’s disease. Sci Transl Med. 2023;15:eabp9352. 10.1126/scitranslmed.abp9352. 36652533 10.1126/scitranslmed.abp9352 PMC13248603

[162] Werner MH, Olanow CW, McGarry A, Meyer C, Kruger S, Klint C, et al. A phase I, randomized, SAD, MAD, and PK study of Risvodetinib in older adults and Parkinson’s disease. J Parkinsons Dis. 2024;14:325–34. 10.3233/JPD-230319. 38251063 10.3233/JPD-230319 PMC10977428

[163] Bloem BR, Macklin EA, Schwarzschild MA. GLP-1 agonists to slow down Parkinson’s progression? The quest continues. Med N Y N. 2025;6:100645. 10.1016/j.medj.2025.100645. 10.1016/j.medj.2025.100645 40220748

[164] Hölscher C. Glucagon-like peptide 1 and glucose-dependent insulinotropic peptide hormones and novel receptor agonists protect synapses in Alzheimer’s and Parkinson’s diseases. Front Synaptic Neurosci. 2022;14:955258. 10.3389/fnsyn.2022.955258. 35965783 10.3389/fnsyn.2022.955258 PMC9363704

[165] Zhang M, Wu Y, Gao R, Chen X, Chen R, Chen Z. Glucagon-like peptide-1 analogs mitigate neuroinflammation in Alzheimer’s disease by suppressing NLRP2 activation in astrocytes. Mol Cell Endocrinol. 2022;542:111529. 10.1016/j.mce.2021.111529. 34906628 10.1016/j.mce.2021.111529

[166] Bu L-L, Liu Y-Q, Shen Y, Fan Y, Yu W-B, Jiang D-L, et al. Neuroprotection of Exendin-4 by enhanced autophagy in a parkinsonian rat model of α-Synucleinopathy. Neurother J Am Soc Exp Neurother. 2021;18:962–78. 10.1007/s13311-021-01018-5. 10.1007/s13311-021-01018-5 PMC8423983 33723752

[167] Cheng D, Yang S, Zhao X, Wang G. The role of glucagon-like peptide-1 receptor agonists (GLP-1 RA) in diabetes-related neurodegenerative diseases. Drug Des Devel Ther. 2022;16:665–84. 10.2147/DDDT.S348055. 35340338 10.2147/DDDT.S348055 PMC8943601

[168] Wu C-C, Islam MM, Lee A-J, Su C-H, Weng Y-C, Yeh C-Y, et al. Association between statin use and risk of Parkinson’s disease: evidence from 18 observational studies comprising 3.7 million individuals. J Pers Med. 2022;12:825. 10.3390/jpm12050825. 35629248 10.3390/jpm12050825 PMC9145914

[169] Al-Kuraishy HM, Al-Gareeb AI, Alexiou A, Papadakis M, Alsayegh AA, Almohmadi NH, et al. Pros and cons for statins use and risk of Parkinson’s disease: an updated perspective. Pharmacol Res Perspect. 2023;11:e01063. 10.1002/prp2.1063. 36811160 10.1002/prp2.1063 PMC9944858

[170] Lin C-H, Chang C-H, Tai C-H, Cheng M-F, Chen Y-C, Chao Y-T, et al. A double-blind, randomized, controlled trial of lovastatin in early-stage parkinson’s disease. Mov Disord Off J Mov Disord Soc. 2021;36:1229–37. 10.1002/mds.28474. 10.1002/mds.28474 33449392

[171] Rubio-Osornio M, León CTG-D, Montes S, Rubio C, Ríos C, Monroy A, et al. Repurposing simvastatin in Parkinson’s disease model: protection is throughout modulation of the neuro-inflammatory response in the substantia Nigra. Int J Mol Sci. 2023;24:10414. 10.3390/ijms241310414. 37445592 10.3390/ijms241310414 PMC10341642

[172] Stevens KN, Creanor S, Jeffery A, Whone A, Zajicek J, Foggo A, et al. Evaluation of simvastatin as a disease-modifying treatment for patients with Parkinson disease: a randomized clinical trial. JAMA Neurol. 2022;79:1232–41. 10.1001/jamaneurol.2022.3718. 36315128 10.1001/jamaneurol.2022.3718 PMC9623477

[173] Riederer P, Nagatsu T, Youdim MBH, Wulf M, Dijkstra JM, Sian-Huelsmann J. Lewy bodies, iron, inflammation and neuromelanin: pathological aspects underlying Parkinson’s disease. J Neural Transm Vienna Austria 1996. 2023;130:627–46. 10.1007/s00702-023-02630-9. 10.1007/s00702-023-02630-9 PMC10121516 37062012

[174] Yao Z, Jiao Q, Du X, Jia F, Chen X, Yan C, et al. Ferroptosis in Parkinson’s disease -- the iron-related degenerative disease. Ageing Res Rev. 2024;101:102477. 10.1016/j.arr.2024.102477. 39218077 10.1016/j.arr.2024.102477

[175] Gonzalez-Alcocer A, Duarte-Jurado AP, Soto-Dominguez A, Loera-Arias MdeJ, Villarreal-Silva EE, Saucedo-Cardenas O, et al. Unscrambling the role of redox-active biometals in dopaminergic neuronal death and promising metal chelation-based therapy for Parkinson’s disease. Int J Mol Sci. 2023;24:1256. 10.3390/ijms24021256. 36674772 10.3390/ijms24021256 PMC9867532

[176] Devos D, Rascol O, Meissner WG, Foubert-Samier A, Lewis S, Tranchant C, et al. Therapeutic modalities of deferiprone in Parkinson’s disease: SKY and EMBARK studies. J Park Dis. 2025;15:72–86. 10.1177/1877718X241300295. 10.1177/1877718X241300295 PMC13347414 39973479

[177] Devos D, Labreuche J, Rascol O, Corvol J-C, Duhamel A, Guyon Delannoy P, et al. Trial of deferiprone in Parkinson’s disease. N Engl J Med. 2022;387:2045–55. 10.1056/NEJMoa2209254. 36449420 10.1056/NEJMoa2209254

[178] Negida A, Hassan NM, Aboeldahab H, Zain YE, Negida Y, Cadri S, et al. Efficacy of the iron-chelating agent, deferiprone, in patients with Parkinson’s disease: a systematic review and meta-analysis. CNS Neurosci Ther. 2024;30:e14607. 10.1111/cns.14607. 38334258 10.1111/cns.14607 PMC10853946

[179] Yang SY, Taanman JW, Gegg M, Schapira AHV. Ambroxol reverses tau and α-synuclein accumulation in a cholinergic N370S GBA1 mutation model. Hum Mol Genet. 2022;31:2396–405. 10.1093/hmg/ddac038. 35179198 10.1093/hmg/ddac038 PMC9307316

[180] Mullin S, Smith L, Lee K, D’Souza G, Woodgate P, Elflein J, et al. Ambroxol for the treatment of patients with Parkinson disease with and without glucocerebrosidase gene mutations: a nonrandomized, noncontrolled trial. JAMA Neurol. 2020;77:427–34. 10.1001/jamaneurol.2019.4611. 31930374 10.1001/jamaneurol.2019.4611 PMC6990847

[181] Colucci F, Avenali M, De Micco R, Fusar Poli M, Cerri S, Stanziano M, et al. Ambroxol as a disease-modifying treatment to reduce the risk of cognitive impairment in GBA-associated Parkinson’s disease: a multicentre, randomised, double-blind, placebo-controlled, phase II trial. The AMBITIOUS study protocol. BMJ Neurol Open. 2023;5:e000535. 10.1136/bmjno-2023-000535. 38027469 10.1136/bmjno-2023-000535 PMC10679992

[182] McHale-Owen H, Faller KME, Chaytow H, Gillingwater TH. Phosphoglycerate kinase 1 as a therapeutic target in neurological disease. Trends Mol Med. 2025;31:1077–88. 10.1016/j.molmed.2025.03.008. 40234116 10.1016/j.molmed.2025.03.008

[183] Cai R, Zhang Y, Simmering JE, Schultz JL, Li Y, Fernandez-Carasa I, et al. Enhancing glycolysis attenuates Parkinson’s disease progression in models and clinical databases. J Clin Invest. 2019;129:4539–49. 10.1172/JCI129987. 31524631 10.1172/JCI129987 PMC6763248

[184] Simmering JE, Welsh MJ, Liu L, Narayanan NS, Pottegård A. Association of glycolysis-enhancing α-1 blockers with risk of developing Parkinson disease. JAMA Neurol. 2021;78:407–13. 10.1001/jamaneurol.2020.5157. 33523098 10.1001/jamaneurol.2020.5157 PMC7851758

[185] Simmering JE, Welsh MJ, Schultz J, Narayanan NS. Use of glycolysis-enhancing drugs and risk of Parkinson’s disease. Mov Disord. 2022;37:2210–6. 10.1002/mds.29184. 36054705 10.1002/mds.29184 PMC9669185

[186] Weber MA, Sivakumar K, Tabakovic EE, Oya M, Aldridge GM, Zhang Q, et al. Glycolysis-enhancing α1-adrenergic antagonists modify cognitive symptoms related to Parkinson’s disease. NPJ Park Dis. 2023;9:32. 10.1038/s41531-023-00477-1. 10.1038/s41531-023-00477-1 PMC9981768 36864060

[187] Liu J-Q, Zhao M, Zhang Z, Cui L-Y, Zhou X, Zhang W, et al. Rg1 improves LPS-induced Parkinsonian symptoms in mice via inhibition of NF-κB signaling and modulation of M1/M2 polarization. Acta Pharmacol Sin. 2020;41:523–34. 10.1038/s41401-020-0358-x. 32203085 10.1038/s41401-020-0358-x PMC7468333

[188] Yang Y, Wang L, Zhang C, Guo Y, Li J, Wu C, et al. Ginsenoside Rg1 improves Alzheimer’s disease by regulating oxidative stress, apoptosis, and neuroinflammation through Wnt/GSK-3β/β-catenin signaling pathway. Chem Biol Drug Des. 2022;99:884–96. 10.1111/cbdd.14041. 35313087 10.1111/cbdd.14041

[189] He Y-B, Liu Y-L, Yang Z-D, Lu J-H, Song Y, Guan Y-M, et al. Effect of ginsenoside-Rg1 on experimental Parkinson’s disease: a systematic review and meta-analysis of animal studies. Exp Ther Med. 2021;21:552. 10.3892/etm.2021.9984. 33850524 10.3892/etm.2021.9984 PMC8027743

[190] Wang S-S, Peng Y, Fan P-L, Ye J-R, Ma W-Y, Wu Q-L, et al. Ginsenoside Rg1 ameliorates stress-exacerbated Parkinson’s disease in mice by eliminating RTP801 and α-synuclein autophagic degradation obstacle. Acta Pharmacol Sin. 2025;46:308–25. 10.1038/s41401-024-01374-w. 39227736 10.1038/s41401-024-01374-w PMC11747340

[191] Hu X, Li J, Wang X, Liu H, Wang T, Lin Z, et al. Neuroprotective effect of melatonin on sleep disorders associated with Parkinson’s disease. Antioxid Basel Switz. 2023;12:396. 10.3390/antiox12020396. 10.3390/antiox12020396 PMC9952101 36829955

[192] Reiter RJ, Sharma RN, Manucha W, Rosales-Corral S, Almieda Chuffa LGde, Loh D, et al. Dysfunctional mitochondria in age-related neurodegeneration: utility of melatonin as an antioxidant treatment. Ageing Res Rev. 2024;101:102480. 10.1016/j.arr.2024.102480. 39236857 10.1016/j.arr.2024.102480

[193] Ribeiro RFN, Santos MR, Aquino M, de Almeida LP, Cavadas C, Silva MMC. The therapeutic potential of melatonin and its novel synthetic analogs in circadian rhythm sleep disorders, inflammation-associated pathologies, and neurodegenerative diseases. Med Res Rev. 2025;45:1515–39. 10.1002/med.22117. 40344229 10.1002/med.22117

[194] Li J, Liu H, Wang X, Xia Y, Huang J, Wang T, et al. Melatonin ameliorates Parkinson’s disease via regulating microglia polarization in a RORα-dependent pathway. NPJ Park Dis. 2022;8:90. 10.1038/s41531-022-00352-5. 10.1038/s41531-022-00352-5 PMC9270337 35803929

[195] Zheng R, Ruan Y, Yan Y, Lin Z, Xue N, Yan Y, et al. Melatonin attenuates neuroinflammation by down-regulating NLRP3 Inflammasome via a SIRT1-dependent pathway in MPTP-induced models of Parkinson’s DISEASE. J Inflamm Res. 2021;14:3063–75. 10.2147/JIR.S317672. 34267535 10.2147/JIR.S317672 PMC8275196

[196] Ahn JH, Kim M, Park S, Jang W, Park J, Oh E, et al. Prolonged-release melatonin in Parkinson’s disease patients with a poor sleep quality: a randomized trial. Parkinsonism Relat Disord. 2020;75:50–4. 10.1016/j.parkreldis.2020.03.029. 32480307 10.1016/j.parkreldis.2020.03.029

[197] Krauss JK, Lipsman N, Aziz T, Boutet A, Brown P, Chang JW, et al. Technology of deep brain stimulation: current status and future directions. Nat Rev Neurol. 2021;17:75–87. 10.1038/s41582-020-00426-z. 33244188 10.1038/s41582-020-00426-z PMC7116699

[198] Neumann W-J, Steiner LA, Milosevic L. Neurophysiological mechanisms of deep brain stimulation across spatiotemporal resolutions. Brain J Neurol. 2023;146:4456–68. 10.1093/brain/awad239. 10.1093/brain/awad239 PMC10629774 37450573

[199] Bucur M, Papagno C. Deep brain stimulation in Parkinson disease: a meta-analysis of the long-term neuropsychological outcomes. Neuropsychol Rev. 2023;33:307–46. 10.1007/s11065-022-09540-9. 35318587 10.1007/s11065-022-09540-9 PMC10148791

[200] Krause P, Reimer J, Kaplan J, Borngräber F, Schneider G-H, Faust K, et al. Deep brain stimulation in early onset Parkinson’s disease. Front Neurol. 2022;13:1041449. 10.3389/fneur.2022.1041449. 36468049 10.3389/fneur.2022.1041449 PMC9713840

[201] Hariz M, Blomstedt P. Deep brain stimulation for Parkinson’s disease. J Intern Med. 2022;292:764–78. 10.1111/joim.13541. 35798568 10.1111/joim.13541 PMC9796446

[202] Yan R, Zheng X, Yin Y, Zhang J, Cui Y, Su D, et al. Treatment for dyskinesia in Parkinson’s disease: a network meta-analysis of randomized controlled trials. Mov Disord Off J Mov Disord Soc. 2025;40:869–80. 10.1002/mds.30179. 10.1002/mds.30179 40099430

[203] Chiu SY, Tsuboi T, Hegland KW, Herndon NE, Shukla AW, Patterson A, et al. Dysarthria and speech intelligibility following Parkinson’s disease globus pallidus internus deep brain stimulation. J Park Dis. 2020;10:1493–502. 10.3233/JPD-202246. 10.3233/JPD-202246 32955467

[204] Pinto S, Nebel A, Rau J, Espesser R, Maillochon P, Niebuhr O, et al. Results of a randomized clinical trial of speech after early neurostimulation in Parkinson’s disease. Mov Disord Off J Mov Disord Soc. 2023;38:212–22. 10.1002/mds.29282. 10.1002/mds.29282 36461899

[205] Krishnan S, Shetty K, Puthanveedu DK, Kesavapisharady K, Thulaseedharan JV, Sarma G, et al. Apraxia of lid opening in subthalamic nucleus deep brain stimulation for Parkinson’s disease-frequency, risk factors and response to treatment. Mov Disord Clin Pract. 2021;8:587–93. 10.1002/mdc3.13206. 33981792 10.1002/mdc3.13206 PMC8088107

[206] Zoon TJC, van Rooijen G, Balm GMFC, Bergfeld IO, Daams JG, Krack P, et al. Apathy induced by subthalamic nucleus deep brain stimulation in Parkinson’s disease: a meta-analysis. Mov Disord Off J Mov Disord Soc. 2021;36:317–26. 10.1002/mds.28390. 10.1002/mds.28390 PMC7986158 33331023

[207] Pagano G, Taylor KI, Anzures-Cabrera J, Marchesi M, Simuni T, Marek K, et al. Trial of prasinezumab in early-stage Parkinson’s disease. N Engl J Med. 2022;387:421–32. 10.1056/NEJMoa2202867. 35921451 10.1056/NEJMoa2202867

[208] Pagano G, Taylor KI, Anzures Cabrera J, Simuni T, Marek K, Postuma RB, et al. Prasinezumab slows motor progression in rapidly progressing early-stage Parkinson’s disease. Nat Med. 2024;30:1096–103. 10.1038/s41591-024-02886-y. 38622249 10.1038/s41591-024-02886-y PMC11031390

[209] Pagano G, Monnet A, Reyes A, Ribba B, Svoboda H, Kustermann T, et al. Sustained effect of prasinezumab on Parkinson’s disease motor progression in the open-label extension of the PASADENA trial. Nat Med. 2024;30:3669–75. 10.1038/s41591-024-03270-6. 39379705 10.1038/s41591-024-03270-6 PMC11645263

[210] Xiao B, Tan E-K. Prasinezumab slows motor progression in Parkinsons disease: beyond the clinical data. NPJ Park Dis. 2025;11:31. 10.1038/s41531-025-00886-4. 10.1038/s41531-025-00886-4 PMC11839931 39971932

[211] Kikuchi T, Morizane A, Doi D, Magotani H, Onoe H, Hayashi T, et al. Human iPS cell-derived dopaminergic neurons function in a primate Parkinson’s disease model. Nature. 2017;548:592–6. 10.1038/nature23664. 28858313 10.1038/nature23664

[212] Sawamoto N, Doi D, Nakanishi E, Sawamura M, Kikuchi T, Yamakado H, et al. Phase I/II trial of iPS-cell-derived dopaminergic cells for Parkinson’s disease. Nature. 2025;641:971–7. 10.1038/s41586-025-08700-0. 40240591 10.1038/s41586-025-08700-0 PMC12095070

[213] Park T-Y, Jeon J, Lee N, Kim J, Song B, Kim J-H, et al. Co-transplantation of autologous Treg cells in a cell therapy for Parkinson’s disease. Nature. 2023;619:606–15. 10.1038/s41586-023-06300-4. 37438521 10.1038/s41586-023-06300-4 PMC12012854

[214] Mansour HM, El-Khatib AS. Exploring Parkinson-associated kinases for CRISPR/Cas9-based gene editing: beyond alpha-synuclein. Ageing Res Rev. 2023;92:102114. 10.1016/j.arr.2023.102114. 37924981 10.1016/j.arr.2023.102114

[215] Yoon HH, Ye S, Lim S, Jo A, Lee H, Hong F, et al. CRISPR-Cas9 gene editing protects from the A53T-SNCA overexpression-induced pathology of Parkinson’s disease in vivo. CRISPR J. 2022;5:95–108. 10.1089/crispr.2021.0025. 35191750 10.1089/crispr.2021.0025

[216] Liu J, Zhu Y, Chen B, Meng Q, Hu P, Chen X, et al. Common and specific effects in brain oscillations and motor symptoms of tDCS and tACS in Parkinson’s disease. Cell Rep Med. 2025;6:102044. 10.1016/j.xcrm.2025.102044. 40154492 10.1016/j.xcrm.2025.102044 PMC12047527

